# Sensorimotor correlates of sit-to-stand in healthy adults

**DOI:** 10.3389/fbioe.2025.1605524

**Published:** 2025-07-25

**Authors:** Caitlin McDonald, John Jairo Villarejo Mayor, Olive Lennon

**Affiliations:** School of Public Health, Physiotherapy and Sports Science, University College Dublin, Dublin, Ireland

**Keywords:** EEG, EMG, corticomuscular coherence (CMC), sit-to-stand (STS), kinematics

## Abstract

**Introduction:**

Standing up, while one of the most common daily activities is also one of the most mechanically demanding tasks undertaken in daily life. Mobility impairments, in particular neurological conditions, often impede individuals’ ability to stand up independently. Despite the obvious association between neurological disorders and impairment of sit-to-stand, the neurophysiological basis of this functional movement is not well understood, particularly at brain level.

**Methods:**

Subjects (N = 20, 4 males) performed fifteen sets of five sit-to-stand transitions on an armless, backless seat adjusted to the knee joint height of each participant. Electromyography (EMG) was recorded from the bilateral vastus lateralis, biceps femoris, tibialis anterior, and gastrocnemius. Surface electroencephalography (EEG) activity was recorded using eight focused bipolar channels over the sensorimotor cortex. Kinematic data was recorded using a three-dimensional motion capture camera system.

**Results:**

EMG and kinematic data confirm distinct flexion and extension phases of the movement with timed co-activation of the quadriceps and hamstrings, and gastrocnemius and tibialis anterior. EEG data demonstrates a change in cortical activity across the phases of sit-to-stand, notably event-related desynchronisation in the higher band frequencies (14–35 Hz) in the flexion and early extension phase, most prominent at the central Cz electrode. Corticomuscular coherence was observed during the flexion and extension phases between the Cz electrode and the biceps femoris and gastrocnemius, in a subgroup of participants.

**Discussion:**

This study provides insights into how cortical activity modulates movement execution during sit-to-stand. The event-related spectral perturbation data contributes to our understanding of this movement by revealing frequency specific changes in cortical activity across the phases of the sit-to-stand transition. Corticomuscular coherence was highest during the flexion phase when transitioning to extension, congruent with electroencephalography and Electromyography activity levels. Whether the brain activity observed is sufficient to distinguish between kinematic phases remains to be determined.

## 1 Introduction

Standing up and sitting down, while two of the most common daily activities (Schenkman et al., 1990; Janssen et al., 2002) are also two of the most mechanically demanding tasks undertaken in daily life (Riley et al., 1991; Carr et al., 2002; Yu et al., 2000). As a necessary precursor to gait, the sit-to-stand transfer is commonly used as a functional, task-specific training activity in rehabilitation (Janssen et al., 2002). Standing up from a seated position is a complex dynamic task that involves generating significant vertical force and requires appropriate postural control to transition from a large to a smaller base of support (Vaughan-Graham et al., 2019). This performance is affected by physiological and psychological factors, including lower-limb strength, lower-limb proprioception, peripheral tactile sensitivity, active hip, knee, and ankle joint range of motion, anxiety and visual contrast sensitivity (Vaughan-Graham et al., 2019; Lord et al., 2002; Kuo et al., 2010).

Older adults and other populations with motor impairments often have difficulties performing sit-to-stand transfers independently (Janssen et al., 2002) and this is associated with a poorer health related quality of life (Davis et al., 2015), increased rates of hospitalisation, risk of falls and death (Volpato et al., 2011; Hirvensalo et al., 2000; Greene et al., 2019). In addition, those at high risk of falls are more likely to fall during sit-to-stand transitions than during walking itself (van Schooten et al., 2018). Neurological disorders often negatively impact the execution of the sit-to-stand transfer, impeding its successful completion. For example, following a stroke, reduced peak vertical reaction force, larger medio-lateral centre of pressure displacement and altered weight distribution in the lower-limbs is observed (Boukadida et al., 2015; Cheng et al., 1998; Pollock et al., 2014). Despite this obvious association between neurological disorders and impairment in sit-to-stand dynamics, the neurophysiological basis of this functional movement is still not fully understood.

The kinematics of sit-to-stand have been comprehensively studied, largely considered as four distinct dynamic phases. Phase one comprising upper body forward momentum initiation, phase two pelvic de-weighting with momentum transfer, phase three upward body extension and phase four erect postural stabilisation (McDonald et al., 2024). Extrinsic factors such as seat height, foot position and the use of armrests can alter hip and knee moments, momentum generation requirements and the movement strategy adopted (McDonald et al., 2024; Sadeh et al., 2023). Electromyography (EMG) activity of the lower-limb muscles during the sit-to-stand movement is also well documented with three primary muscle synergies reported (Hanawa et al., 2017). During the momentum transfer phase, Tibialis Anterior muscles are mostly activated, followed by Quadriceps (Hanawa et al., 2017). During the extension phase, Quadriceps, Hamstrings, and Gluteus Maximus muscles are co-activated and in the stabilisation phase soleus and gastrocnemius are mainly activated (Hanawa et al., 2017). These distinct muscle synergies remain evident even in the presence of central neurological pathologies like stroke and Parkinson’s disease (McDonald et al., 2024).

However, to fully understand the neurophysiological bases of the complex sit-to-stand movement, electroencephalography (EEG) data must also be considered, integrated and studied collectively with kinematic and EMG data (Kim et al., 2017) to allow the neural top–down control of physiological function to be understood. For example, when the coherence between brain motor cortex and associated muscles (corticomuscular coherence) is critically examined (Liu et al., 2019). In gait analysis, corticomuscular coherence (CMC) confirms that the motor cortex actively controls the lower limb muscle activity and forward propulsion during both overground and treadmill walking (Artoni et al., 2017; Jensen et al., 2019). In stark contrast, limited knowledge of brain activity during sit-to-stand transfers exists. A recent systematic review of the neural basis of sit-to-stand identified no published studies that recorded and reported EEG data during 3D kinematic capture of the movement (McDonald et al., 2024). Under-exploration of EEG activity during sit-to-stand activity is due, in part, to practical limitations that include significantly higher EEG artefact during complex movements like sit-to-stand, limitations in older wire based systems and movements where muscles of the face and neck are active (Kline et al., 2021). As a result, EEG research during sit-to-stand has focused mostly on imagined movement; classification of different lower limb movements that include standing up and identification of the intention to stand up prior to execution (McDonald et al., 2024). Existing data reporting brain activity during sit-to-stand transitions are more limited but do identify beta wave (14–17 Hz) activity in the cerebral cortex of healthy adults during the gross activity, irrespective of the distinct movement phases (Kanokwan et al., 2019).

Therapeutic robotics in rehabilitation is a rapidly growing field (Chang and Kim, 2013) with brain machine interface applications that decode motor intent and/or activity a major Frontier. Efforts to date have been largely focussed on the upper limbs (Camargo-Vargas et al., 2021) or animal based studies of lower-limb activities (Ma et al., 2017). Some promising EEG based classification studies (Bulea et al., 2013; Bulea et al., 2014; Jochumsen and Niazi, 2020; Chang et al., 2023) have recognised the intention to stand up or sit with up to 81% accuracy in a closed loop application based on dynamical region connectivity and entropy of EEG (Chang et al., 2023). This points to meaningful data in EEG related to standing intent, with greater accuracy in intent detection with co-registered EEG and EMG data for hybrid- Brain Computer Interface (BCI) (Yang et al., 2022; Sherwani and Khan, 2022). Predicting the phases or motor trajectory of sit-to-stand using EEG and/or EMG may be more critical to the development of responsive robotic rehabilitation devices however (Lennon et al., 2020) Therefore further data is required to establish the neurophysiological bases of the complex sit-to-stand movement, integrating EMG, EEG and kinematic data (Kim et al., 2017). The aim of this study is to investigate cortical activity during sit-to-stand transitions by analysing synchronised EEG, EMG, and kinematic data using event-related spectral perturbation (ERSP) and corticomuscular coherence (CMC) across different phases of the movement.

## 2 Materials and methods

### 2.1 Participants

Participants were volunteers by self-selection recruited through poster campaigns on a university campus and in local retirement groups. Participants were included if they were over 18 years of age, had no known neurological condition and could stand independently from a regular height surface without upper limb support. Informed and written consent was obtained from each subject prior to participation. The Human Research Ethics Committee at University College Dublin, the National University of Ireland approved the experimental protocols (LS-22-19-Lennon), conducted in accordance with the Declaration of Helsinki.

### 2.2 Data collection

**FIGURE 1:**
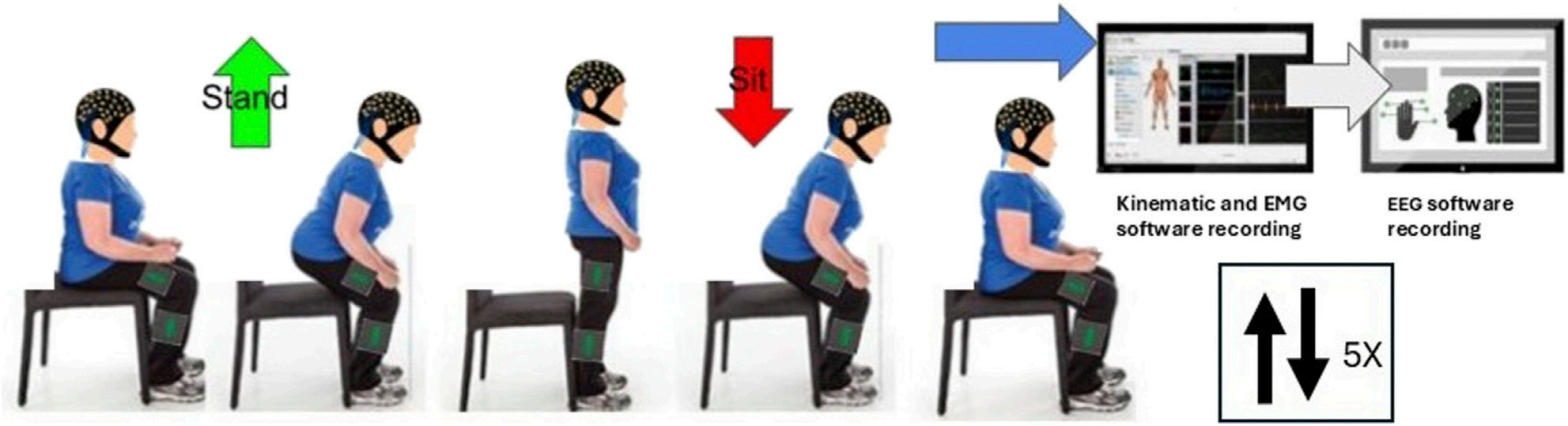
Data Collection protocol.

Figure 1 details the data collection protocol. Participants were positioned on an armless, backless seat, adjusted in height to the knee joint height of each participant with the knee at 90° flexion. Initially a 2-min resting EEG trial was recorded. This was followed by a target of fifteen sets of five sit-to-stand and stand-to-sit transitions at a self-selected pace, pausing for approximately 5 s between each transition. Subjects were instructed to stand up and sit down in a natural way, without using upper limb assistance. To minimise movement artefact in EEG recordings, subjects were asked to avoid excessive blinking, teeth grinding and jaw clenching. To ensure familiarity with the task and consistency across subjects, participants performed a practice trial of 5 sit-to-stand repetitions with verbal correction or demonstrations provided where required.

**FIGURE 2:**
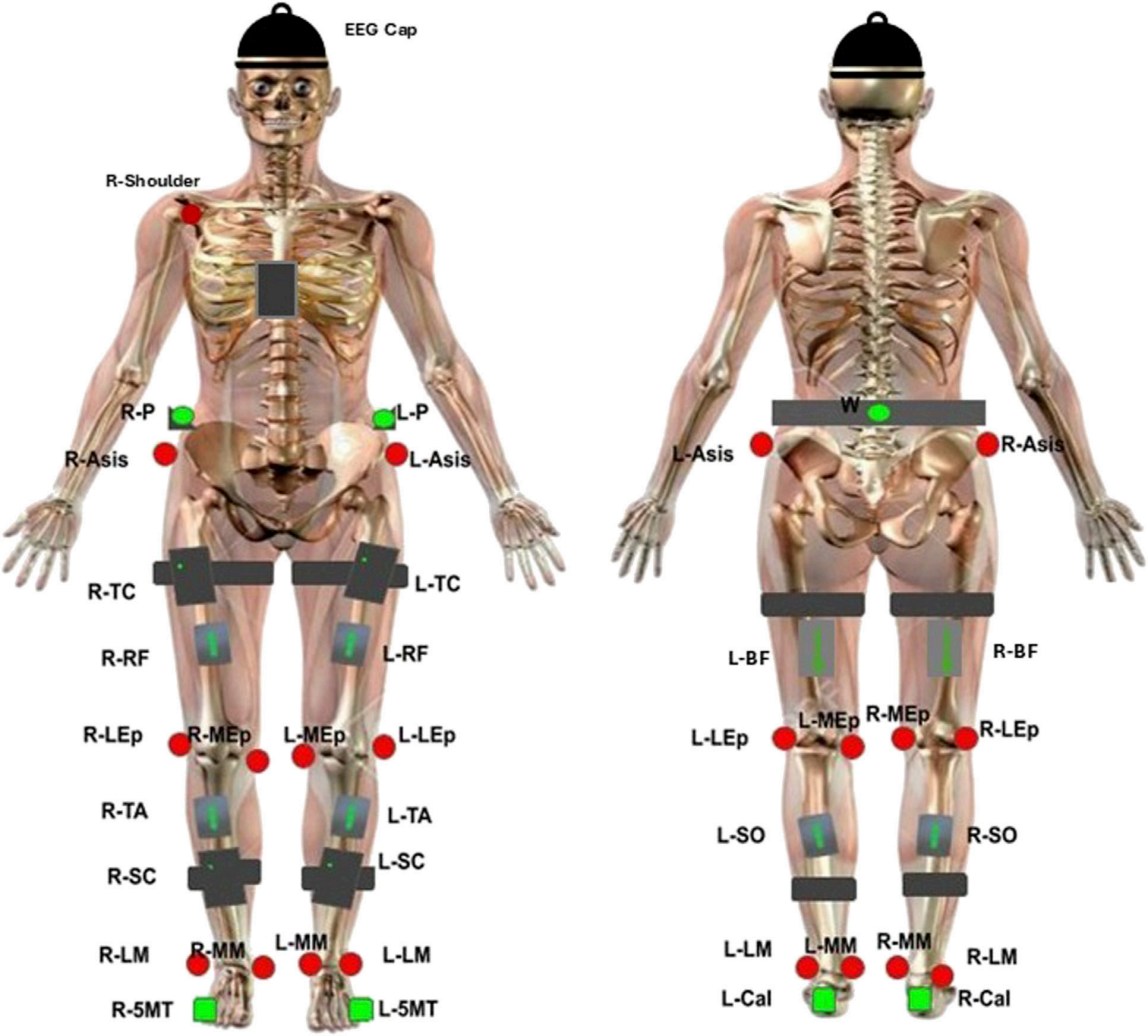
EEG, EMG and kinematic sensor placement. Green circles represent single-active CODAmotion sensors. Black boxes represent CODAmotion cluster markers. Red circles represent virtual markers generated by CODAmotion software. Grey boxes represent wireless Delsys EMG sensors. The NeuroCONCISE Flex EEG Cap is worn.

Figure 2 depicts data collection equipment used. EEG was recorded at a sampling frequency of 250 Hz using a wireless system of eight bipolar channels (FlexEEG, NeuroCONCISE, United Kingdom) over the sensorimotor cortex [C6, C4, C2, Cz, FCz, C1, C3, and C5 locations defined by the international 10/20 system of electrode placement (Klem et al., 1999)]. Electrode Afz was used as a reference. Conductive gel was used to maintain low electrode impedance. Data were recorded and saved using Matlab software (Mathworks, United States).

Electrical muscle activity was measured using eight wireless EMG electrodes with a 2000-Hz sampling rate (Delsys, Trigno, United States). Electrodes were placed bilaterally on vastus lateralis (VL), biceps femoris (BF), tibialis anterior (TA), and gastrocnemius (GAS) muscle groups according to SENIEM guidelines (Stegeman and Hermens, 2007).

A 3D motion capture camera system was used to record the kinematic joint angles (Codamotion 3D system, United Kingdom). Four cameras captured the movement by active infrared retroreflective wearable marker clusters, attached to both sides of the body, on the pelvis, upper leg, lower leg, calcaneus, and fifth metatarsal bases, and one marker cluster was placed in the centre of the chest. In addition, reflective markers were attached bilaterally to anatomical landmarks (anterior superior iliac spine, lateral femoral epicondyle, and medial femoral epicondyle; lateral and medial malleoli) and the right acromioclavicular joint, to create a three-dimensional whole-body segment model. Kinematic data were recorded at a 100-Hz sampling rate. Hip, knee and ankle joint angles were estimated from spatial position data by the Codamotion ODIN software platform (ODIN, Codamotion, United Kingdom).

All EEG, EMG and ODIN data were synchronised. EMG data was integrated digitally with the kinematic data via Coda-Hub. Synchronisation of the FlexEEG system with Coda-Hub was achieved using a Delsys Trigger Module (Delsys, United States) and a LabJack U12 USB DAQ module (LabJack, United States). When kinematic recording began, a pulse from the trigger module was sent to Coda-Hub to start EMG recording, and a second pulse was sent from Coda-Hub to Matlab (Mathworks, United States) through the LabJack module via USB.

### 2.3 Data processing

The sit-to-stand movement was divided into two broad dynamic phases based on kinematic joint angle data (collapsing the four known phases of sit-to-stand to two). Phase one, the flexion phase, began with initial detection of hip flexion movement and ended at maximum hip flexion (combining upper body forward momentum and pelvic de-weighting phases). Phase two, the extension phase, began at maximum hip flexion and ended at maximum hip extension (combining the extension and stabilisation phases). Pre and post movement activity were recorded in quiet sitting, 0.5 s prior to the beginning of the flexion phase, and in upright standing 0.5 s after termination of the extension phase.

EMG data were bandpass filtered between 20 and 250 Hz (zero-phase 2nd -order Chebyshev). A 50 Hz notch filter was used to remove power-line interference. The offset for each channel was removed by subtracting the trial signal average.

Raw EEG were bandpass filtered, 0.1–60 Hz with a zero-phase 2nd -order Chebyshev. A notch filter centred at 50 Hz with a 2 Hz bandwidth was used to remove power line interference. EEG data were then submitted to an automated artefact rejection procedure, Artificial Space Removal (ASR) using the EEGLAB toolbox (clean_artifacts) in Matlab (Mathworks, United States) to remove artefacts related to low frequency drifts, noisy channels and short-time bursts (Delorme and Makeig, 2004; Chang et al., 2019; Kothe and Jung, 2016). For each subject, ASR was calibrated from the EEG data collected during the 2-min resting state condition and applied to all the movement trials performed, in line with previous studies (Artoni et al., 2017; Tortora et al., 2020; Tortora et al., 2023). The resting period was used instead of the more common task-specific period as the short trial duration and movement artifact limited calibration. Segments with absolute voltages above 100 µV were removed, as these values typically indicate the presence of non-neural artifacts such as muscle activity or movement-related artifact. Following pre-processing, time-domain EEG signals were visually inspected to identify and exclude channels contaminated by artifacts.

Pre-processed EEG data underwent manual visual inspection in the time and frequency domain. Channels with high level of artefact that were deemed not to contain meaningful EEG information were excluded from analysis (Table 2). Epochs that lacked characteristic EEG features such as well-defined oscillatory patterns and consistent baseline activity were excluded. Signals with abrupt, irregular deflections, non-physiological flat-lining, high-frequency noise inconsistent with neural activity were flagged as likely artifacts and discarded. This manual inspection process ensured the retention of only physiologically plausible EEG data for further analysis.

### 2.4 Data analysis

Data sets were individually screened for quality of EEG, EMG and Kinematic signals. In the case where one of the three inputs was not viable or deemed not to be of sufficient quality, that entire sit-to-stand trial was discarded. Participants were excluded from analysis if they did not reach a minimum of 20 viable sit-to-stand transitions. EEG signal quality across time course were inspected. If an individual channel did not contain meaningful EEG information that single channel was removed from analysis.

ERSP was calculated over the movement phases, to measure the modulation of the EEG activity in the frequency domain across sit-to-stand phases. The time-frequency decomposition was computed on each channel and trial and then averaged across trials at each frequency. For normalisation, the mean power across the full-task duration was computed for each frequency and used as a baseline. This approach is appropriate for dynamic motor tasks, to highlight time-varying spectral modulations relative to the task average (Tortora et al., 2023; Gwin et al., 2010). This analysis was performed using the EEGLAB toolbox (newtimef) (Delorme and Makeig, 2004), providing the epoch times at which the phases occur in each trial using the argument “timewarp”, and setting the “trialbase” argument to “full” to perform single trial normalisation. These settings were used to align the timepoints of each phase for the different trials. ERSP plots masked for non-significant features (p > 0.05) were also computed.

Power spectral density (PSD) was computed from the ERSP for each phase of sit-to-stand. Topographic maps were generated using the EEGLAB toolbox (topoplot) (Delorme and Makeig, 2004) applying a mask for the surrounding canals (T7, T8, Fz, Pz, P4, P3, F3, and F4). Theta (4–8 Hz), alpha (8–12 Hz), low-beta (12–18 Hz), high-beta (18–30 Hz) and gamma (30–40 Hz) power bands were calculated and normalised to their average power. As described, the typical beta frequency band (12–18 Hz) was divided into low-beta and high-beta bands, as this had been identified as a frequency range of interest in previous sit-to-stand and gait studies (Tortora et al., 2023; Mustile et al., 2024; Roeder et al., 2020).

Pre-processed EMG data were normalised on an individual basis in amplitude to the maximum value found within each repetition (sets of five trials) to reduce the variability across subjects. An envelope was obtained by the Root Mean Square (RMS) using 250 ms sliding windows, overlapping 50 ms. EMG signal of each phase was timewarped using linear interpolation (0.1% resolution), to align the timepoints for averaging the different trials.

For CMC analysis, we examined the frequency-domain coupling between the EEG electrode Cz, which is located over primary motor cortical area, and each of the eight recorded leg muscles. We chose this centrally located sensor because it is widely employed to assess CMC during gait (Jensen et al., 2019; Gennaro and de Bruin, 2020; da Silva Costa et al., 2024) and it was found to show maximal beta-band CMC amplitudes (Spedden et al., 2019). Preliminary analysis, performed between the C1, C2, C3, C4, C5, C6 and FCz electrodes and each muscle respectively, also confirmed the Cz electrode provided the most information when calculating CMC during sit-to-stand. On account of individual trial duration of the sit-to-stand phases being too short to allow for reliable spectral analysis, EMG and EEG during all trials were concatenated for each phase, as an alternative to averaging, to improve the spectral resolution (Johnson and Shinohara, 2012; Gangwani et al., 2023). A low pass filter, 100 Hz with a zero-phase 2nd -order Chebyshev, was then applied on both EMG and EEG data. EMG data was full-wave rectified prior to coherence analysis. The Matlab coherence function, with a Hamming window of 512 and overlapping of 192 samples, was used for CMC calculation. The plots were masked for non-significant features (p > 0.05). The significance of the coherence was assessed though surrogate data analysis, generating 75 surrogate coherence spectra between random signals for every subject, preserving the properties by using the same length of the original signals (Faes et al., 2004). A cumulative density distribution with the surrogate coherence peaks were built to compare against the real coherence peaks for statistical significance (p < 0.05) (Piitulainen et al., 2015).

A Pearson correlation analysis was conducted to evaluate the relationship firstly, between age and presence of significant CMC between Cz and at least one muscle, and secondly between the number of trials analysed and presence of CMC between Cz and at least one muscle. Both were coded as 1 for positive presence of CMC and 0 for negative presence of CMC. The significance level was set at p < 0.05.

## 3 Results

A total of twenty-three healthy volunteers completed the data collection protocol. As detailed in Table 1, three participants’ data were excluded from analysis based on dataset viability issues. Data from 20 subjects (4 males) with a mean age of 43 ± 19.2 years, height 173 ± 8.4 cm and weight 74.3 ± 10.3 kg are presented. A total of 1,140 sit-to-stand repetitions were included across 20 participants were included in the analysis. Further detail on included trials and any electrodes removed from subsequent analysis are detailed in Table 2.

**TABLE 1:** Participant demographics.

Participant	Sex (M/F)	Age (years)	Height (cm)	Weight (kg)
1	F	25	180	64
2	F	69	171	63.5
3	F	25	173	73
4	F	26	173	73
5	F	55	174.5	78
6	F	25	165	76
7	F	35	172	100
8	M	24	186	85
9	F	28	165	64
10	F	25	180.3	72
11	M	30	177	63
12	F	70	162	78
13	F	75	165	64
14	F	70	160.5	88
15	F	38	166.5	66
16	F	26	167	69
17	M	32	192	86
18	F	19	178	59
19	F	27	173	73
20	M	42	172	64
	6 Male/17 Female	40.38 ± 19.19 years	173.37 ± 7.68 cm	73.8 ± 9.91 kg

**TABLE 2:** Dataset details.

Participant	Number of sit-to-stand transitions	Channels excluded due to artefact^a^
1	75	—
2	50	Cz, C1, C5
3	60	—
5	75	C6
6	75	—
7	65	—
8	30	Cz for 15 trials
10	20	C2
11	70	C1, FCz
12	40	Cz, C4, C6
13	30	—
14	70	Cz
15	35	—
17	70	—
18	65	—
19	45	C3, C5
20	70	C2 for 65 trials
21	75	C6
22	75	—
23	45	—

^a^ Channels excluded from all trials unless specified.

**FIGURE 3:**
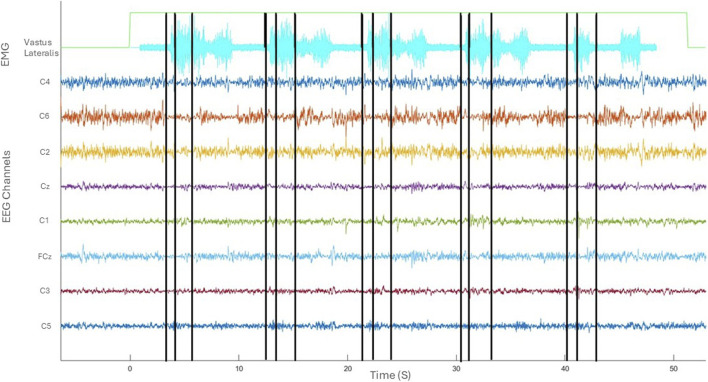
Co-registered kinematics, EMG activity of vastus lateralis and filtered EEG of one subject in the time domain (seconds).

Figure 3 provides an overview of the co-registered data depicting one trial of five sit-to-stand and stand-to-sit transitions. The kinematic phases of sit-to-stand are illustrated by black lines; the first marking the beginning of the flexion phase, the second the beginning of the extension phase and the final line is the end of the movement. A sample of EMG activity from the right vastus lateralis is shown and filtered EEG data from the eight sensorimotor channels. Results are next presented separately broken down by kinematics, muscle (EMG) activity, cortical (EEG) activity and corticomuscular coherence during the movement phases.

### 3.1 Kinematics

**FIGURE 4:**
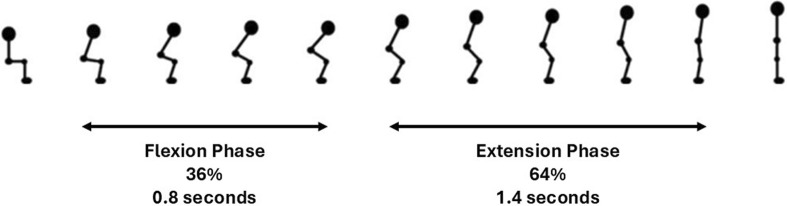
Kinematics of sit-to-stand transfer.

The average time for a participant to complete the sit-to-stand transfer was 2.2 s. The sit-to-stand transfer when considered as two dynamic movement phases, comprised 36% (0.8 s, SD 0.28 s) as the flexion phase and 64% (1.4 s, SD 0.48 s) as the extension phase. In the early flexion phase the hips and ankles began to flex to initiate the movement. Hip flexion continued and the hip was unloaded from the seat with some continuing knee and ankle flexion evident. In the extension phase the hips and knees began to extend, coinciding with ankle plantar flexion. Towards the end of the extension phase as the body sought stability in upright standing, there are small joint adjustments seen largely at the ankle joint. This is illustrated in Figure 4.

### 3.2 EMG

**FIGURE 5:**
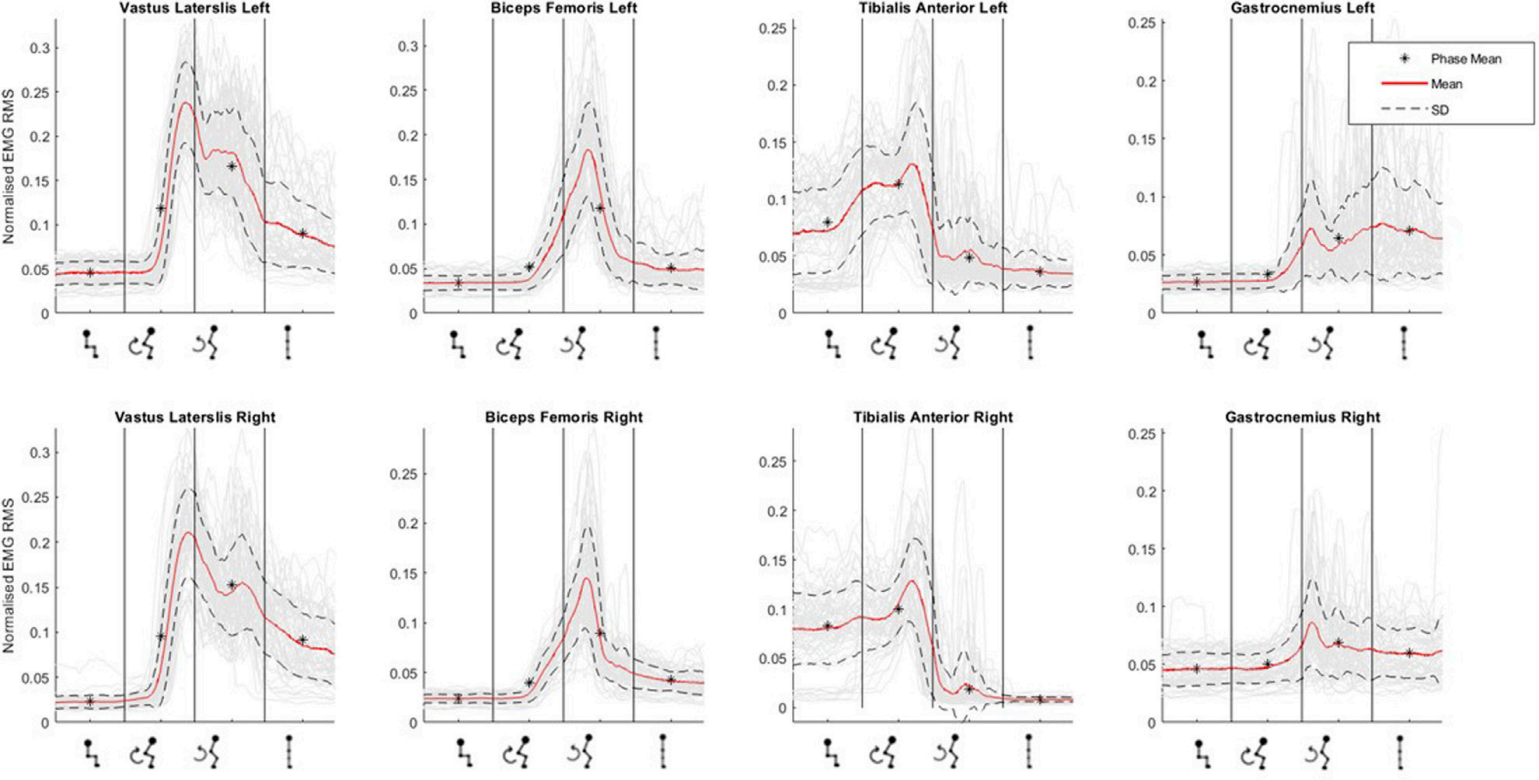
Muscle activation (as normalised EMG RMS) across phases of sit-to-stand (sitting prior to movement, flexion phase, extension phase and upright standing) averaged across all participants.

Figure 5 graphically depicts the average lower-limb muscle activity (EMG RMS) of the four lower-limb muscles across sit-to-stand movement phases. Tibilalis anterior is first activated in the flexion phase, closely followed by vastus lateralis and biceps femoris. All three muscles reach peak EMG activity at seat off, the moment when the hip is unloaded from the chair and the hip joint angle trajectory changes from flexion to extension, i.e., the transition point between the flexion and extension phases. Across these three muscle groups, activity then reduces until upright standing is achieved, when activity plateaus marginally above (∼0.5 RMS) baseline sitting activity. The gastrocnemius is most active in the extension phase and remains active in upright standing. The muscle that reaches the highest level of normalised RMS activity is the vastus lateralis. Minor inter-limb differences were observed in peak EMG activity across all muscle groups in individual subjects.

### 3.3 EEG

To evaluate cortical involvement during the sit-to-stand transition, event-related spectral perturbation (ERSP) across the sit-to-stand phases was first calculated, measuring the relative modulation of the EEG activity in the frequency domain when standing-up.

**FIGURE 6:**
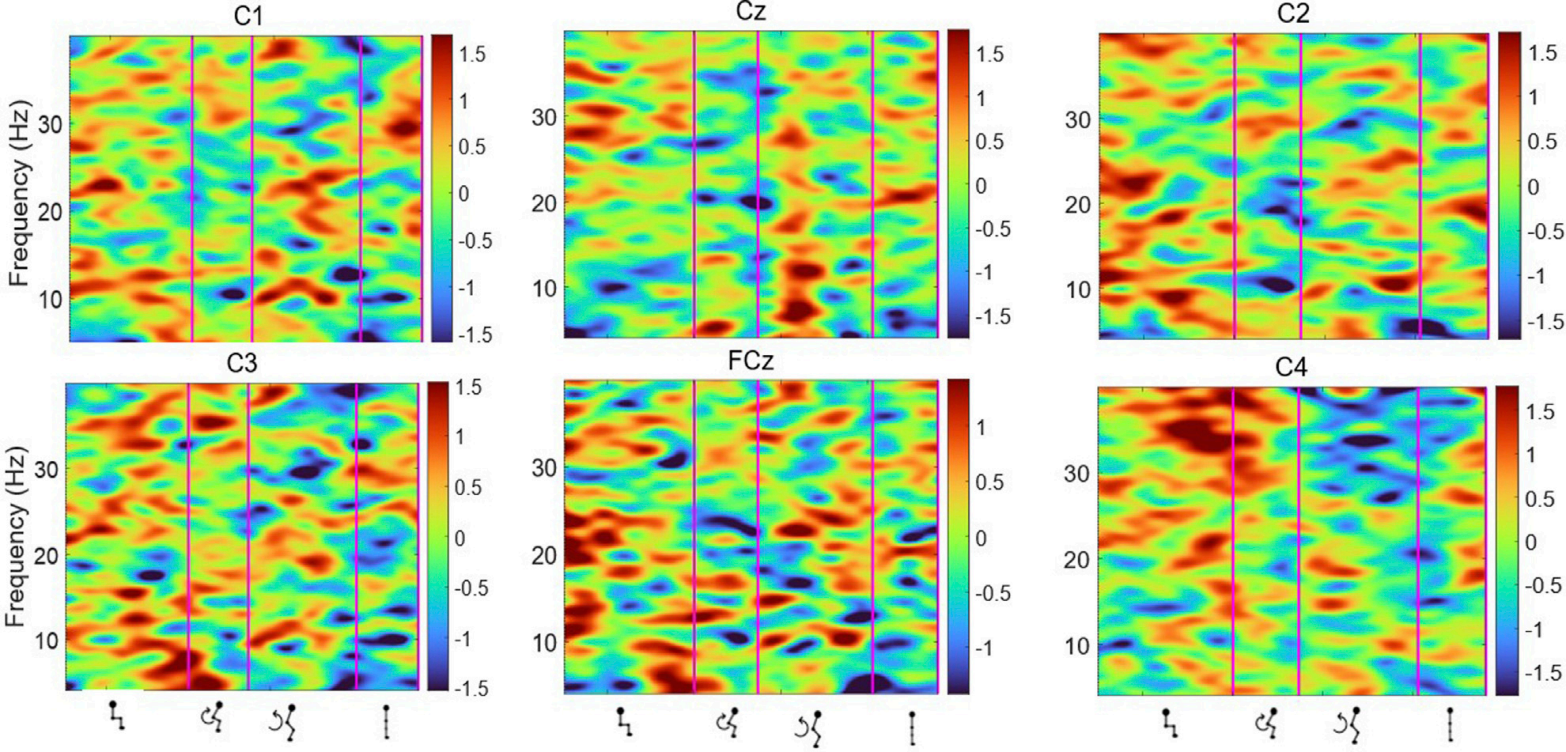
Average ERSP output for the Cz, FCz, C1, C2, C3 and C4 channels over the sit-to-stand transition, across all 75 trials for one subject (Schenkman et al., 1990). Y-axis frequency between 4 and 40 Hz, and in x-axis the time in ms and kinematic phases of standing up. Red and yellow spots represent synchronisation, and darker blue spots represent desynchronisation.

**FIGURE 7:**
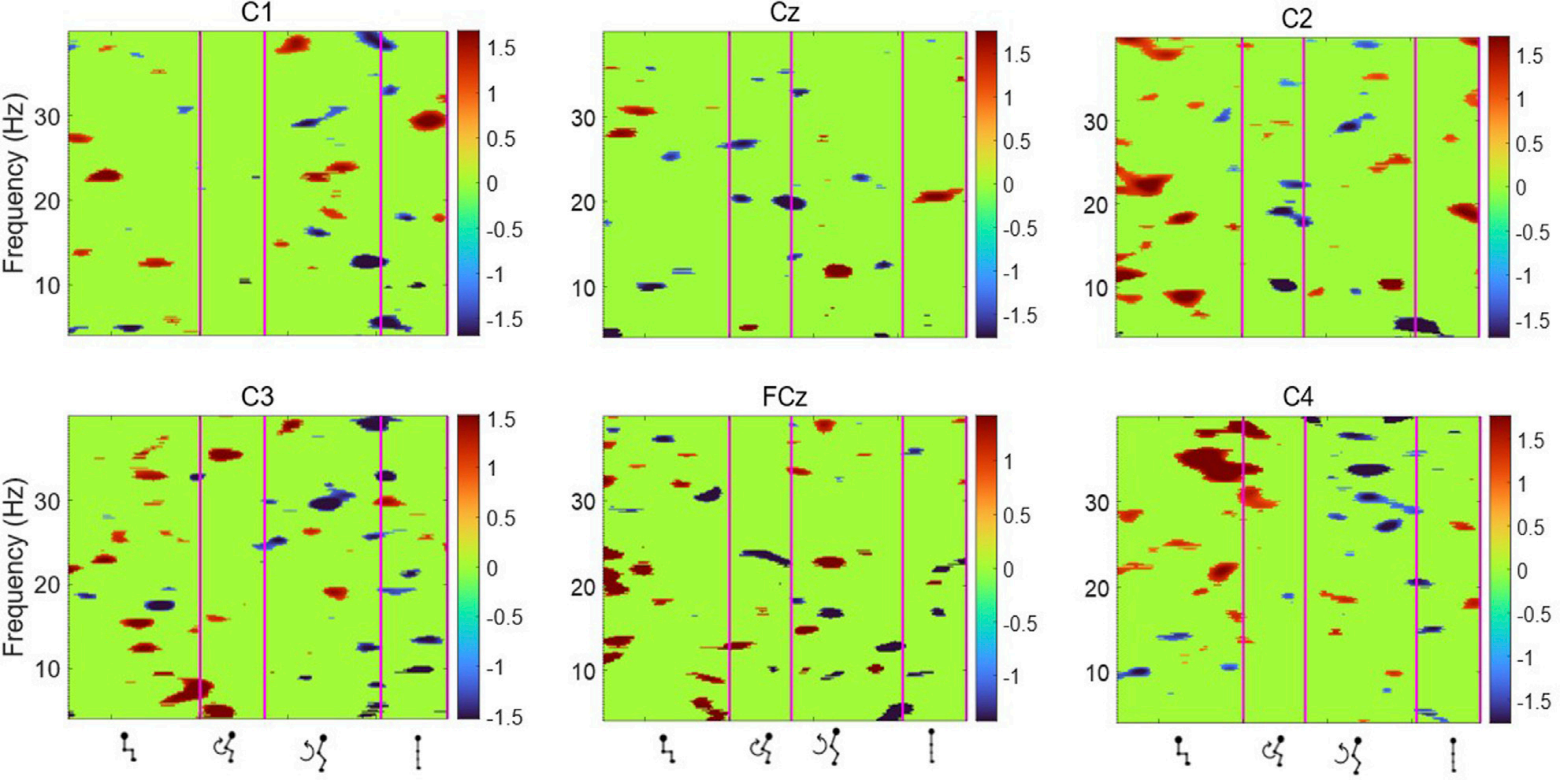
Average ERSP output for the Cz, FCz, C1, C2, C3 and C4 channels across all 75 trials for one participant. Y-axis frequency between 4 and 40 Hz, and in x-axis the time in ms and kinematic phases of standing up. ERSP plots are masked for significance (p < 0.05, in green).

**FIGURE 8:**
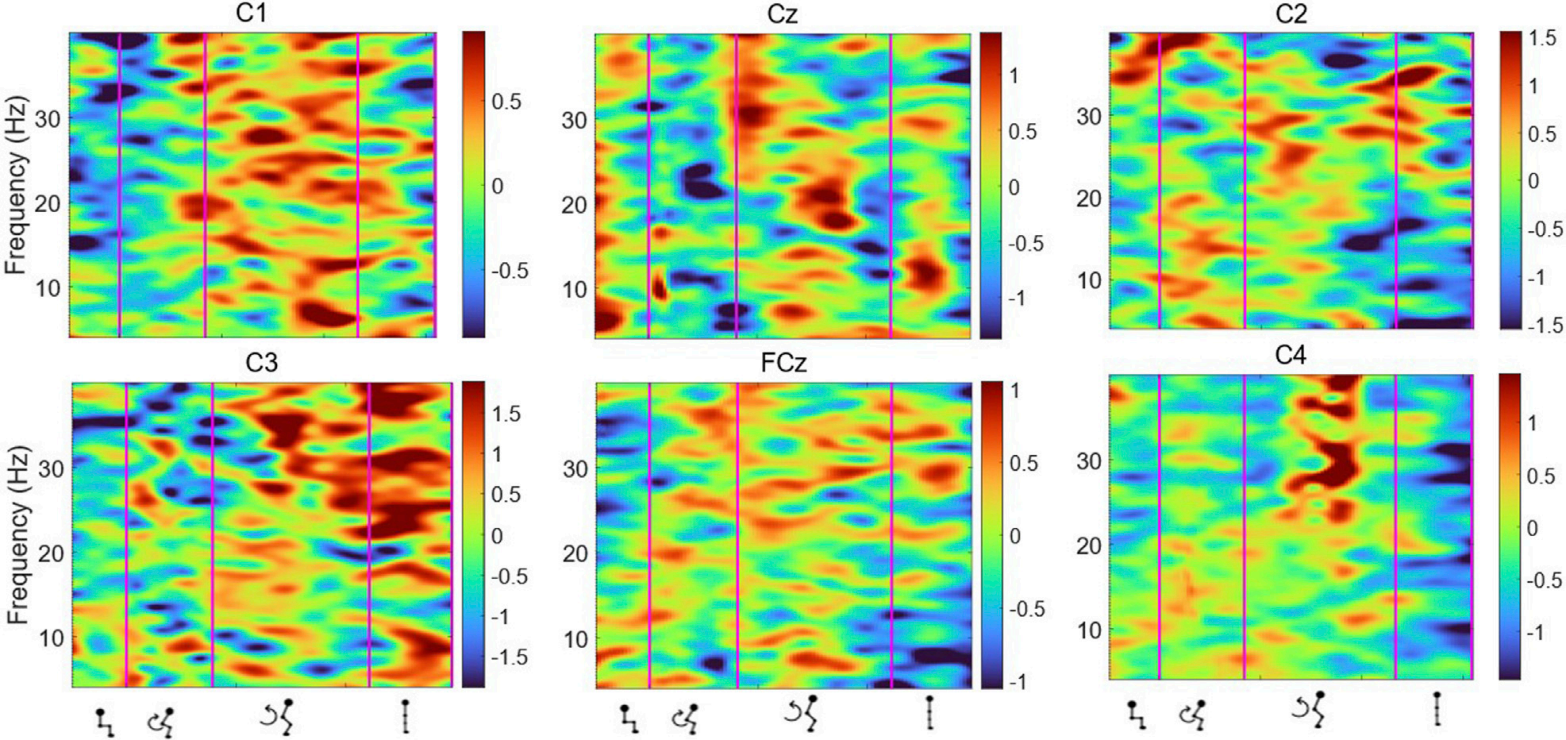
Average ERSP output for the Cz, FCz, C1, C2, C3 and C4 channels across all trials for all participants. Y-axis frequency between 4 and 40 Hz, and in x-axis the time in ms and kinematic phases of standing up. Red and yellow spots represent synchronisation, and darker blue spots represent desynchronisation.

Figures 6, 7 display the ERSP in the time-frequency domain for one individual (subject 1), as per the phases of sit-to-stand. Pre-movement sitting (0.5 s) and steady standing on termination of the sit-to-stand transfer (0.5 s) are also depicted. Individual ERSP plots for each subject are included in the Supplementary Material. Compared to the quiet sitting state, a transient loss in amplitude (Event Related Desynchronisation (ERD)) is seen across high-alpha, beta and gamma frequencies (10–40 Hz) prior to movement onset and in the flexion and early extension phase. De-synchronisation continues in the Beta and Gamma bands (14–40 Hz) until the participant achieves steady upright standing, when event-related synchronisation (ERS) is seen. This pattern is most pronounced in the midline Cz channel. A similar pattern of synchronisation/de-synchronisation is seen in Figure 8 where a grand average of ERSP spectrogram across all participants is presented. Notably, the grand average shows beta ERD occurring earlier in C1 and C2 than the midline Cz channel.

**FIGURE 9:**
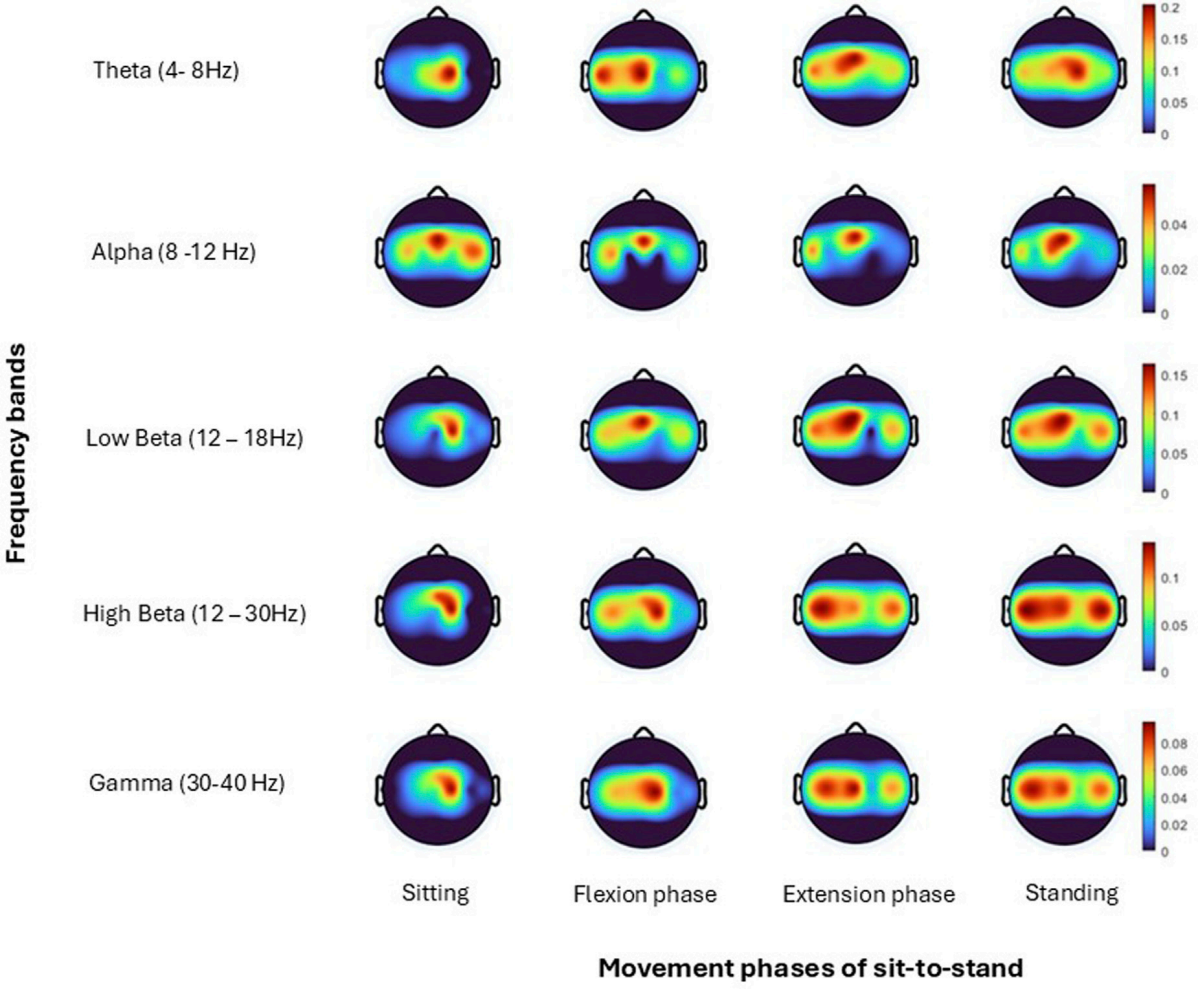
Topoplots show brain activity, across each frequency band (Theta, Alpha, Low Beta, High Beta, Gamma) averaged across all trials for one subject (Hanawa et al., 2017) for each; sitting prior to movement, flexion phase, extension phase and upright standing.

**FIGURE 10:**
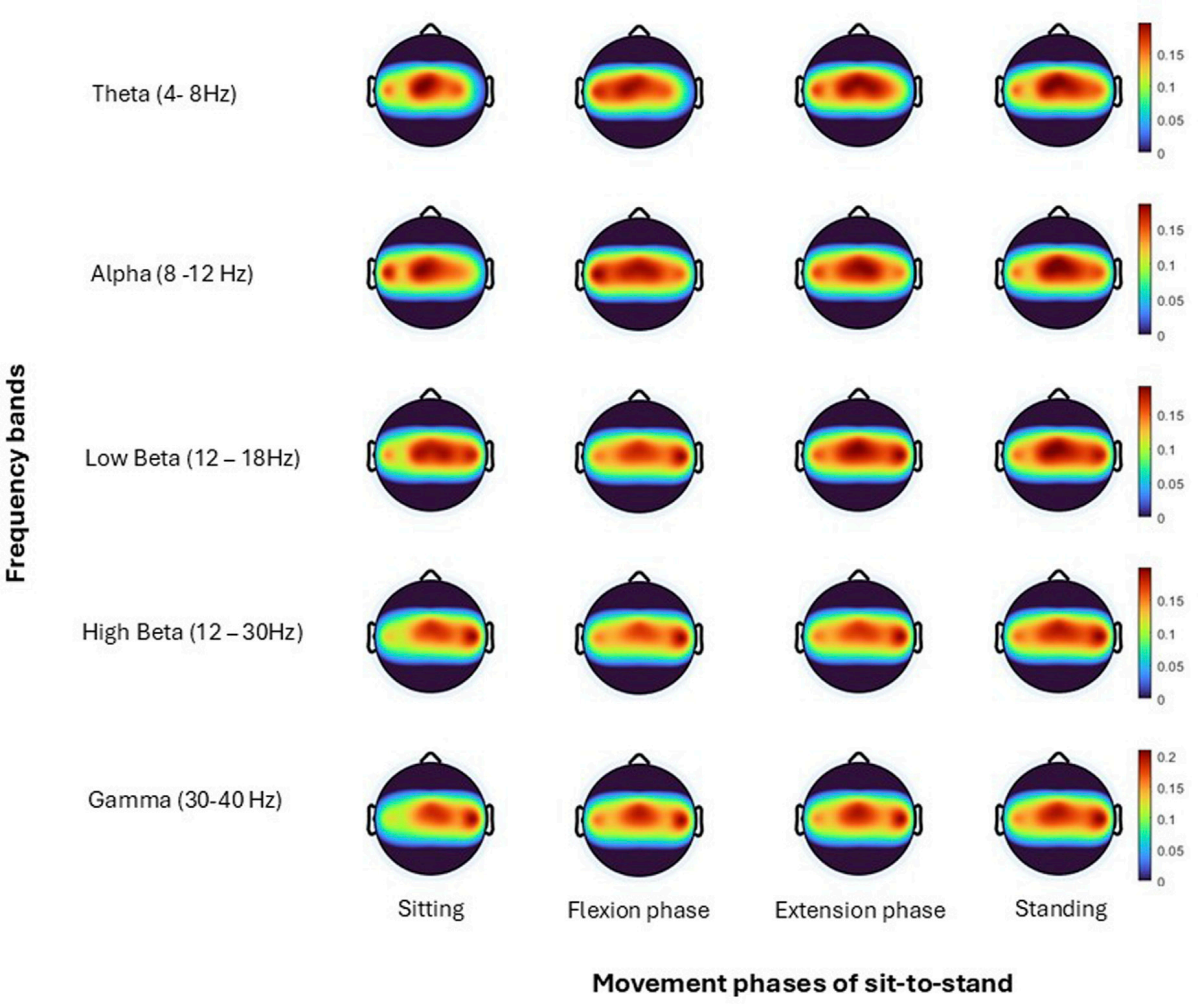
Topoplots show brain activity, across each frequency band (Theta, Alpha, Low Beta, High Beta, Gamma) averaged across all trials for all subjects for each; sitting prior to movement, flexion phase, extension phase and upright standing.

**FIGURE 11:**
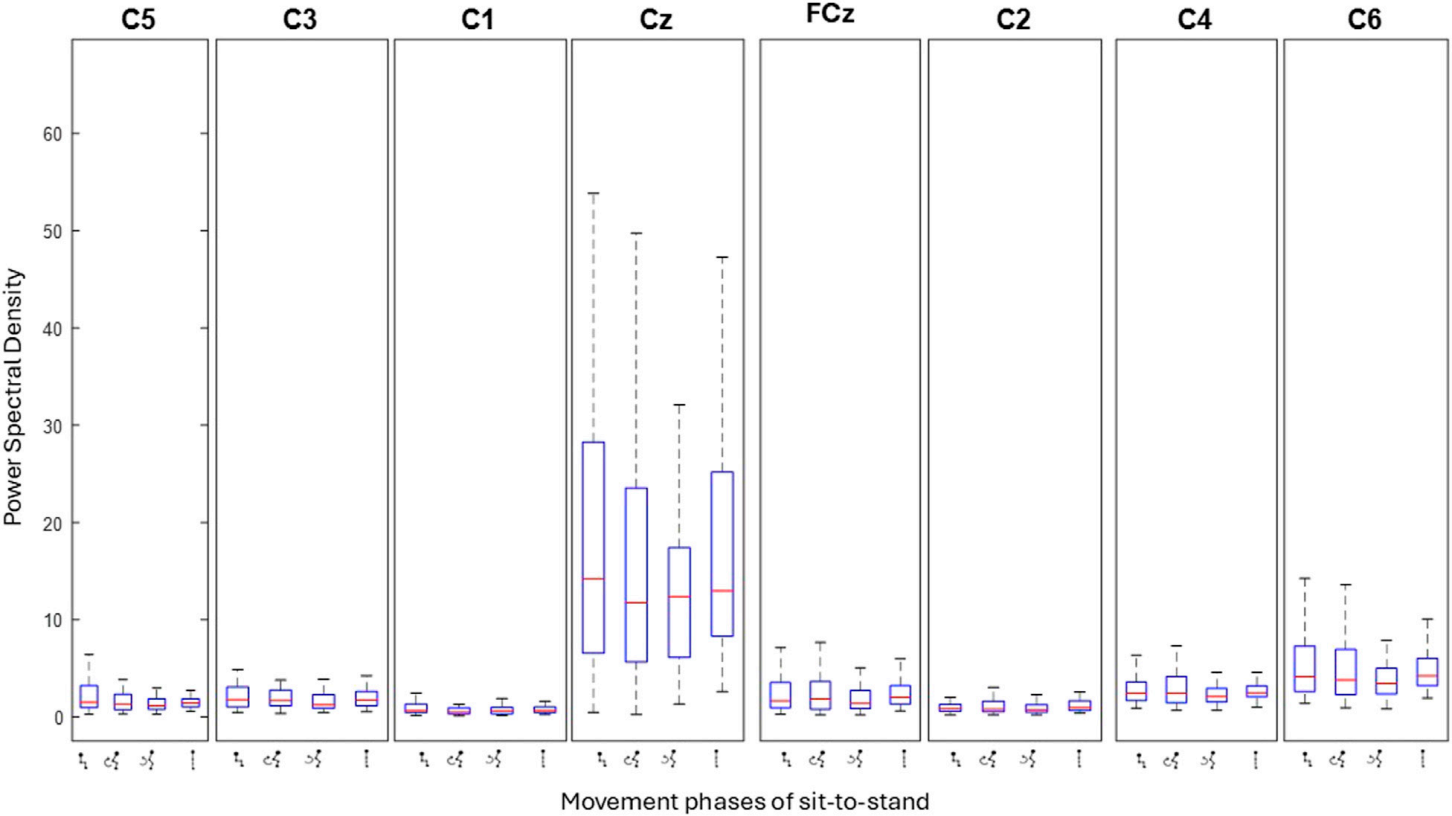
Bar plots show the level of activity (power spectral density) at each electrode across the four phases of sit-to-stand averaged across all trials of one subject (Schenkman et al., 1990). Each box represents individual movement phases.

Topographical mapping of the ERSP spectrogram was next configured to identify the cortical area(s) and electrode(s) which give the most meaningful information during sit-to-stand. EEG is described in terms of rhythmic activity divided into five frequency bands, theta (4–8 Hz), alpha (8–12 Hz), low beta (12–18 Hz), high beta (18–30 Hz) and gamma (30–40 Hz). Results of one participant (Figure 9), and then a grand average across all participants (Figure 10) are presented. The results, which include data in pre-movement sitting and in standing on termination of the sit-to-stand transfer, identify frequency modulation over the sensorimotor cortex. In the single participant example (Figure 9), the Cz location displays alpha and beta desynchronisation during premovement sitting and the flexion phase, alpha desynchronisation in the extension phase, beta synchronisation in the extension phase, and alpha and beta synchronisation in standing. Alpha and beta synchronisation is depicted at the FCz site across the movement phases. While pre-movement sitting showed C2 synchronisation and C1 desynchronisation in the Theta, Beta and Gamma frequencies. A grand average of ERSP spectrogram across all participants does not provide meaningful information related to cortical activity (Figure 10). Diffuse synchronisation is observed pre-movement and across all movement phases. Across the movement phases of sit-to-stand the brain areas that demonstrate the most activity is at the central Cz electrode, followed by the C6 electrode. This is consistent in the Delta, Theta, Alpha, Beta and Gamma frequency bands, as displayed in Figure 11. Additional detail can be found in Supplementary Figure S1.

### 3.4 CMC

**FIGURE 12:**
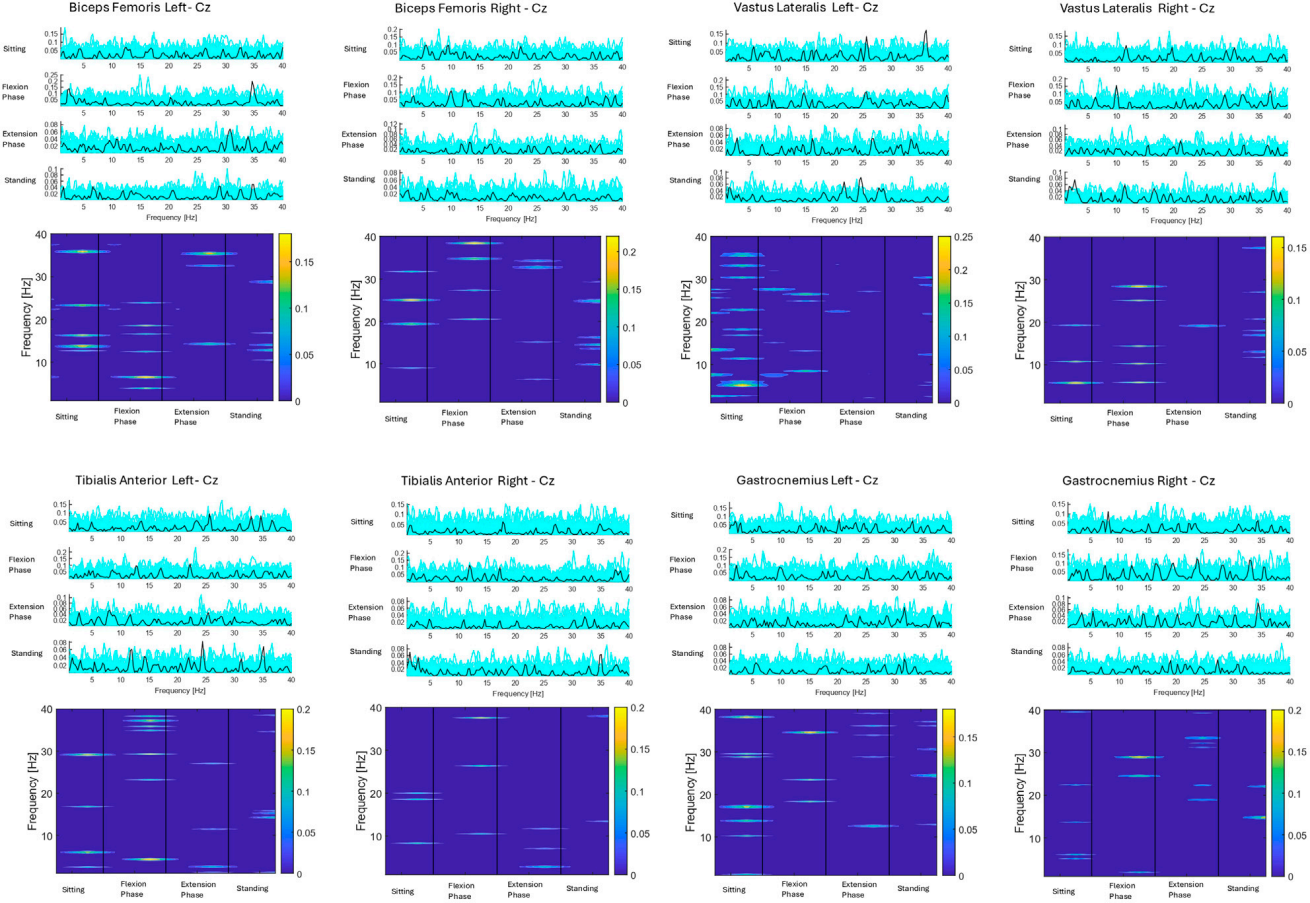
Corticomuscular coherence between the EEG recording at the Cz electrode and EMG recording of the vastus lateralis, biceps femoris, tibialis anterior and gastrocnemius muscles respectively across the phases of sit-to-stand. Data were in all cases pooled data from all repetitions from one subject (Vaughan-Graham et al., 2019) (75 trials). The upper plots show CMC across the frequency domain (black line), alongside 75 randomly generated surrogate coherence spectra (turquoise line). The lower plots show the colour coded magnitude of coherence [masked for non-significant features (p > 0.05)] as indicated using colour bars to the right of the plots.

CMC between the most active (Cz) electrode and individual lower limb muscles (Vastus Lateralis, Biceps Femoris, Tibialis Anterior, Gastrocnemius Lateralis) are depicted for seventeen subjects averaged across all 75 trials. Three subjects did not have viable EEG data from the Cz electrode, as detailed in the methods. Colour plots depict higher coherence in the latter half of the flexion phase and in the extension phase across all muscle groups and all participants consistent with ERSP and EMG findings. However, as detailed in the methods section and presented visually in Figure 12, when CMC is plotted for each phase of sit-to-stand across frequency bands, alongside 75 randomly generated surrogate coherence spectra, the findings do not consistently exceed the chance of identifying corticomuscular coherence from randomly generated data. CMC (p < 0.05) was observed between the Cz electrode and Biceps Femoris in five subjects, three subjects in the flexion phase and two subjects in the extension phase. CMC (p < 0.05) was observed between the Cz electrode and gastrocnemius in four subjects during the extension phase. One subject displayed CMC (p < 0.05) between the vastus lateralis and Cz and one subject between tibialis anterior and Cz, both in the extension phase. CMC plots for all seventeen individual subjects are included in the Supplementary Material.

A Pearson correlation analysis was conducted to evaluate the relationship between age and presence of significant CMC between at least one muscle. The analysis yielded a correlation coefficient of r = −0.110, with a p-value of 0.646. This indicates a weak negative association between age and test result, suggesting a slight, non-significant trend toward younger individuals displaying CMC in at least one muscle. However, the relationship was not statistically significant.

Pearson correlation analysis was next performed to assess the relationship between presence of CMC between at least one muscle and the number of trials. The analysis yielded a correlation coefficient of r = 0.327 with a p-value of 0.20. Although this indicates a moderate positive association, suggesting that positive test results may be associated with a higher number of trials, the relationship was not statistically significant (p > 0.05). Therefore, no conclusive evidence of a meaningful correlation between presence of CMC and number of trials were found.

For the subject presented below statistically significant coherence (p = 0.03) was observed in the flexion phase, between the Cz electrode and the left biceps femoris. The next highest coherence recorded in this subject was between the Cz electrode and the right vastus lateralis (0.28), however this was not statistically significant.

## 4 Discussion

This study, to the authors knowledge, is the first to comprehensively co-register and report 3D kinematic, EMG and EEG data capture during sit-to-stand transfers. The kinematic and EMG data support the wider literature that confirms distinct flexion and extension phases of the movement with timed co-activation of the quadriceps and hamstrings, and gastrocnemius and tibialis anterior (McDonald et al., 2024; Hanawa et al., 2017; Yoshida et al., 2019; Coghlin and McFadyen, 1994). The EEG data, notably the ERSP and topoplot data add new knowledge to our understanding of this movement highlighting a change in cortical activity across the phases of sit-to-stand, notably in the higher band frequencies (between 14 and 35 Hz). Corticomuscular coherence was observed during the late flexion phase and extension phase (when muscle activity was highest) between the Cz electrode and the biceps femoris and the gastrocnemius respectively, in a subset of subjects.

### 4.1 Frequency modulation characteristics

This study presents the first exploration of cortical activity across distinct phases of the sit-to-stand transfer. Results highlight movement induced modulation of the beta rhythm during sit-to-stand, confirming preliminary data (Kanokwan et al., 2019). This was not unexpected, as beta modulation is typically observed in the execution of voluntary movements. However, a major contribution of this study is the exploration of activity across distinct phases of the movement. Here, in preparation to stand up and in the flexion and early extension phases, a loss in beta amplitude is observed (ERD) (Fry et al., 2016) that is followed by an amplitude rebound above baseline [Event-Related Synchronisation (ERS)] or “overshoot” when the upright standing is achieved (Toro et al., 1994; Pfurtscheller, 1992). Consistent with previous EEG literature in the upper limb that identifies power recovery and overshoot happens sequentially, with the higher frequencies recovering earlier (Toro et al., 1994), a more rapid return to baseline values was noted in this study in the 20–30 Hz (high beta) frequency range. While this frequency modulation pattern has previously been observed in other whole body movements such as gait (Borhanazad et al., 2024) and perturbed upright standing (Nakamura et al., 2021) this is the first time data is presented for the complex task of sit-to-stand. Despite the fact that the Beta ERD response elicited is relatively low in amplitude (0.1–0.3 std), as is typical in lower limb movements (Toro et al., 1994), this preliminary data points towards the feasibility of detecting the intention to stand-up from Beta band activity. These findings provide promise for BCI based training of the sit-to-stand activity for those with motor impairments.

### 4.2 Somatotopy of sit-to-stand

The precise, contralateral somatotopic lay out of the motor cortex, as illustrated by the homunculus poses challenges in EEG data collection (Nguyen and Duong, 2023) particularly for the leg area of the motor cortex which is 1–4 cm below the brain surface, vertical in orientation and located at the horizonal fissure (Kline et al., 2021). Lower limb activity captured by the central Cz electrode therefore may capture lower limb activity but cannot distinguish between right and left leg activity. It was not surprising to find increased activity at the Cz electrode during sit-to-stand in this study, although this has not been reported to date in the scientific literature. Results are similar to that described in studies analysing EEG during gait (Jensen et al., 2019; Petersen et al., 2012).

Asymmetry of sit-to-stand in terms of muscle activity amplitude and timing is evident in the literature in healthy individuals and is not necessarily linked to leg dominance, although conflicting reports do exist (Schofield et al., 2014; Burnett et al., 2011; Schofield et al., 2013). It is interesting to note that most participants in the current study displayed increased activity (synchronisation) in the channels of one hemisphere of the brain and minor inter limb differences in EMG peak activity. For example, subject 6, showed increased synchronisation activity in the right midline channels C2 and C4 compared to the contralateral side (C1 and C3). Conversely, their peak EMG activity was higher in the right lower limb muscles compared to their left. This may constitute trunk and/or upper limb counterbalance to weight transfer, but further research is needed to determine the cortical control of this asymmetry.

Participants displayed a high level of activity at the C1 and C2 electrode sites with desynchronisation at these sites occurring earlier than at the central Cz location, potentially indicating activation of the upper limbs early during the sit-to-stand movement. While the protocol was designed to minimise upper limb use; an armless chair was used, participants were instructed to stand up in their natural manner and to avoid using their arms to push off the seat, findings suggest some individuals did use their upper limbs to generate momentum or to maintain balance (Lord et al., 2002; Ng et al., 2015). This is also observed in gait literature where arm swing during natural walking is accompanied by considerable ipsilateral cortical activation (Borhanazad et al., 2024).

Increased activity was also observed in the lateral electrodes C3 and C4. Again, these findings were not unanticipated, given the significant trunk activity during the forward lean and extension phases of standing up (Kılınç et al., 2023; Pourahmadi et al., 2019). While these findings generate new knowledge during the active phases of the movement; previous work by Sherwani and Khan (2022) identified the C4 electrode as providing the most reliable information for classifying the intention to stand-up (Sherwani and Khan, 2022).

### 4.3 CMC

The findings in this review suggest that robust, consistent CMC is not a primary feature of the dynamic sit-to-stand task in healthy adults, or that the methods employed using current surface EEG technology are not sensitive enough. No studies to date have reported CMC during the dynamic sit-to-stand movement (McDonald et al., 2024). Current studies on CMC during isokinetic muscle contractions are limited with the majority of research focusing on isometric contraction, known to produce higher levels of coherence (Liu et al., 2019). CMC has been further shown to be lower in posturally focused, position-control tasks such as sit-to-stand than in force-control tasks (Poortvliet et al., 2015). The complexity of multi-muscle coordination may also hinder the identification of consistent corticomuscular coherence patterns. During sit-to-stand the co-activation of muscle groups occurs resulting in an overlap in time and frequency, effectively diluting the spectral peaks observed in isolated muscle activation (Klous et al., 2010; Farmer et al., 1997). This reduces the signal‐to‐noise ratio of individual muscles EMG recordings but also increases the likelihood that coherence peaks arise spuriously from crosstalk or common input rather than true cortico‐spinal coupling (Halliday et al., 1999; Grosse et al., 2002). Future work should therefore consider using an increased number of electrodes to facilitate more advanced signal‐separation techniques (e.g., independent component analysis or beamforming), or increase sample size to bolster statistical power (Roeder et al., 2020).

Despite these challenges, data presented in this study does suggest weak coherence may be evident in a subset of healthy controls, largely drawn from the coherence levels identified and the consistency between the peak activity levels observed in EEG and EMG and CMC data, potentially indicating inherent biological variability. Higher coherence is consistently observed during the latter half of the Flexion Phase across all muscle groups and participants, particularly in the bilateral biceps femoris. One hypothesis is that the latter half of the flexion phase, immediately before seat off, is when hamstring muscle activity most closely resembles isometric activity. Notably, isometric muscle activity is when the coherence between the cortex and muscle is observed to be at its highest (Liu et al., 2019). This finding, when considered in tandem with the peak muscle activity occurring at this transition phase between flexion and extension phases of sit-to-stand, may warrant consideration clinically where sit-to-stand is often utilised for task specific training (Janssen et al., 2002). Data presented in this study suggest that the transition phase from flexion to extension phases of sit-to-stand may be the component of the movement to focus on for both muscle strengthening and to drive cortical neuroplasticity required to increase independent function of the movement itself.

Although capturing a broad age range is important in order to draw conclusions that extend to the general population by capturing age related variability in physiological, cognitive, or behavioural responses, it is reasonable to assume that the wide age range (43 ± 19.2 years) provided a source of variability in prevalence of significant CMC. Although the present study detected no correlation between the presence of CMC and age (p = 0.65), it has been proven during gait that low beta CMC is reduced in older adults compared to their younger counterparts (Roeder et al., 2020).

Given that significant corticomuscular coherence (CMC) was observed in only a small number of subgroups (e.g., n = 5 for the biceps femoris and n = 4 for the gastrocnemius, out of 17 total subgroups), the study’s limited sample size should be acknowledged as a constraint on the ability to reliably detect consistent CMC patterns. This is the case also for the number of repetitions performed by each participant. Although a high number of repetitions (Witham et al., 2011) were performed by each participant some trials were discarded due to EEG data viability issues, as outlined in the methods. A moderate positive relationship was found between the number of included trials and the presence of significant CMC, indicating participants who displayed significant CMC tended to have a higher number of usable trials, however the correlation was not statistically significant (r = 0.33, P = 0.2). It has been highlighted previously in gait literature that due to the transient and non-stationary nature of EEG/EMG signals during the movement, a high number of gait cycles are required to average out movement-related noise and artifacts to reveal stable CMC estimates (Petersen et al., 2012; Witham et al., 2011). While there is no strict minimum number of repetitions required to calculate CMC, it is possible that the required number is higher during sit-to-stand due to the increased level of EEG artifact during the forward lean phase.

### 4.4 EEG quality considerations

Capturing high quality EEG data during sit-to-stand in the current study was not without its limitations, most notably movement and neck muscle activity artefacts. Mobile EEG, while better than wired systems and allowing free movement, is still susceptible to movement artefact. Therefore, interpreting the phasic changes in EEG activity reported in this study must be approached with judicious caution. In this study, a number of data sets were excluded due to poor quality EEG containing high levels of artefact. While this attention to quality signals was required, it limited our sample size to twenty participants. Individual subjects’ high-quality data were presented alongside grand average data to better verify results. Previous studies have highlighted high levels of EEG artefact during full body movements. Muscle activity artefact was of particular concern due to the activation of the neck extensors in preparation for and during the forward lean of sit-to-stand. EEG artefact related to head and neck muscles can be high, evident even at rest and is compounded by a high number of single muscle units (Yilmaz et al., 2014). Activation of the neck extensors could be responsible for increased artefact at the C5 and C6 sites in particular, due to the neck’s representation in the motor homunculus. These required careful management in this study that included use of bipolar electrodes and a local reference point (AFz), a recorded pre-movement resting baseline and, as detailed in the methods section, a decomposition method that was employed that removed EMG components from raw EEG signals. Future research could consider collecting additional EMG of the neck muscles to quantify their influence and aid in artefact removal.

One strength of the study protocol was the use of targeted bipolar electrodes over the motor sensory cortex which streamlined protocol set up and provided a realistic possibility for donning future BCI devices. However, the relatively small number of EEG channels recorded did limit the separation of artefact sources and the possible mixing of brain and artefact sources cannot be out ruled. Nevertheless acquisition of a larger number of EEG channels still goes not guarantee artefact free EEG signals (Kline et al., 2015). While high-density EEG (HD-EEG) has proven to be the EEG montage that estimates brain activity with highest accuracy, the configurations are often selected to uniformly cover the entire head and not according to their contribution to estimation accuracy. Multiple studies have reported the effect of lowering electrode numbers while retaining accuracy by identifying optimal scalp location and coverage (Soler et al., 2022). A 2022 optimisation-based study to identify the optimal combinations of electrodes that retain the localisation accuracy of HD-EEG reconstructions and found optimal subsets with low-density EEG systems (e.g., consumer-grade wearable EEG such as Neuroconcise) using six electrodes can attain an equal or better accuracy than HD-EEG (with more than 200 channels) (Soler et al., 2022).

## 5 Conclusion

Important information detailing brain activity during kinematic phases of sit-to-stand is evident in this study that warrants further investigation. Results highlight movement induced modulation in specific frequency bands that were largely consistent across phases and participants. The central Cz electrode showed the highest levels of activity with ERD evident during the phases of sit-to-stand. Computation of CMC identified highest coherence during the flexion phase before transitioning to extension, congruent with EMG activity levels. CMC (p > 0.05) was observed between the Cz electrode and the biceps femoris and gastrocnemius respectively, in a subset of subjects. Whether the brain activity observed in this study is sufficient to distinguish between kinematic phases remains to be determined. To develop BCI control of a robotic-assisted sit-to-stand device, additional research is required to further our understanding and determine if the findings reported in this study are consistent across populations with neurological pathologies.

## Data Availability

The original contributions presented in the study are included in the article/Supplementary Material, further inquiries can be directed to the corresponding author.
