# Supplementary material for: Sensorimotor correlates of sit-to-stand in healthy adults
Source: Front Bioeng Biotechnol. 2025 Jul 25;13:1605524. doi: 10.3389/fbioe.2025.1605524 (PMC12331691; doi:10.3389/fbioe.2025.1605524)
Supplement: Supplementary file 1 [file DataSheet1.pdf]

Figure S1

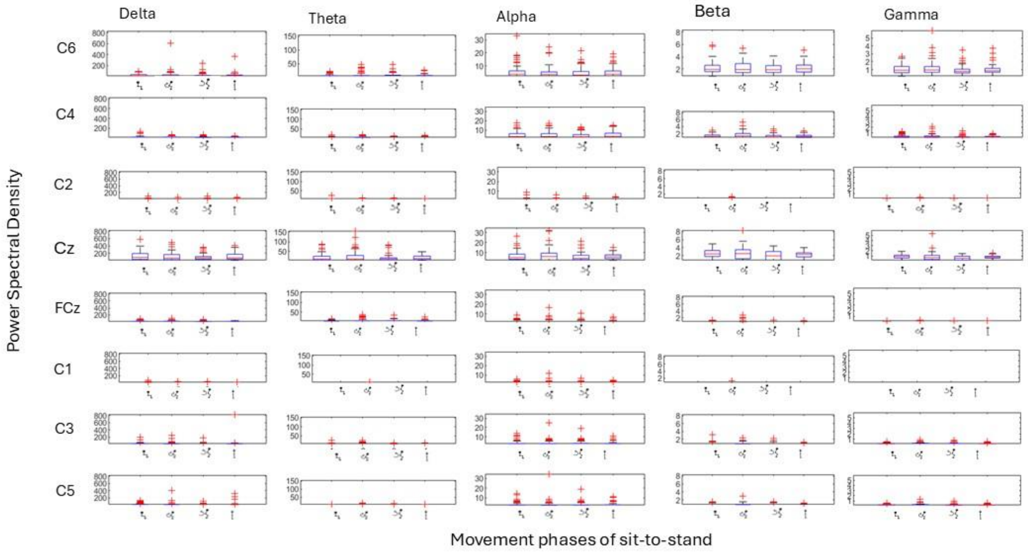

Figure S2

Subject 1: Topoplot

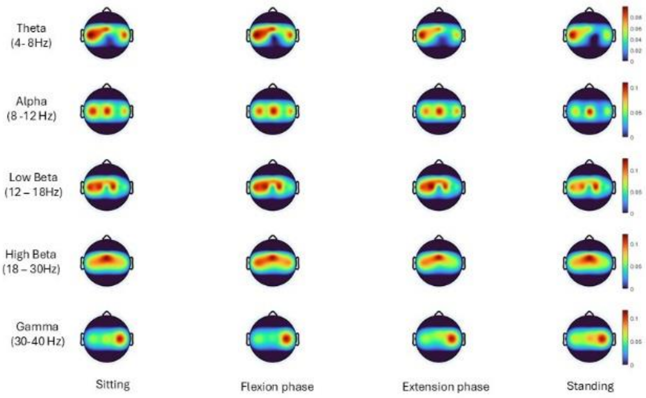

Figure S3

Subject 1: CMC

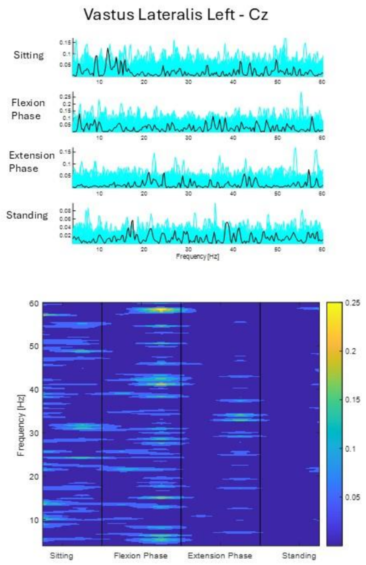
Vastus Lateralis Left - Cz
Sitting
Flexion Phase
Extension Phase
Standing
Frequency [Hz]
Sitting
Flexion Phase
Extension Phase
Standing

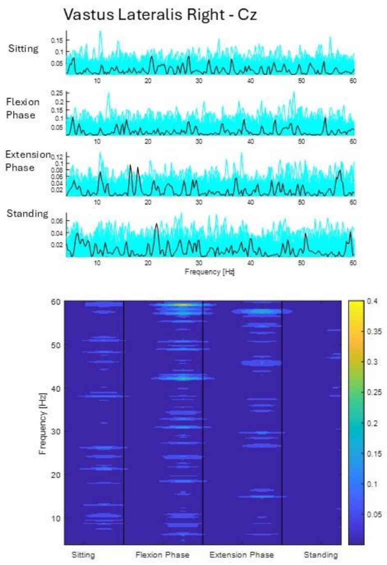
Vastus Lateralis Right - Cz
Sitting
Flexion Phase
Extension Phase
Standing
Frequency [Hz]
Sitting
Flexion Phase
Extension Phase
Standing

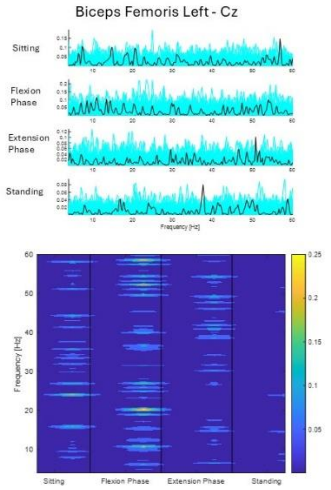
Biceps Femoris Left - Cz
Sitting
Flexion Phase
Extension Phase
Standing
Frequency [Hz]
Sitting
Flexion Phase
Extension Phase
Standing

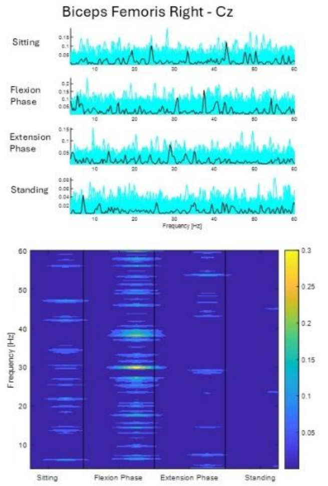
Biceps Femoris Right - Cz
Sitting
Flexion Phase
Extension Phase
Standing
Frequency [Hz]
Sitting
Flexion Phase
Extension Phase
Standing

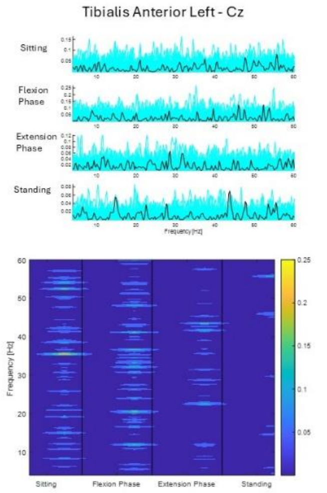
Tibialis Anterior Left - Cz
Sitting
Flexion Phase
Extension Phase
Standing
Frequency [Hz]
Sitting
Flexion Phase
Extension Phase
Standing

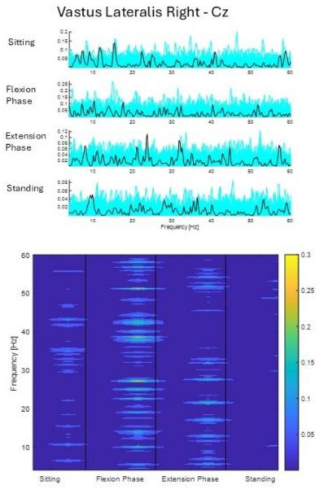
Vastus Lateralis Right - Cz
Sitting
Flexion Phase
Extension Phase
Standing
Frequency [Hz]
Sitting
Flexion Phase
Extension Phase
Standing

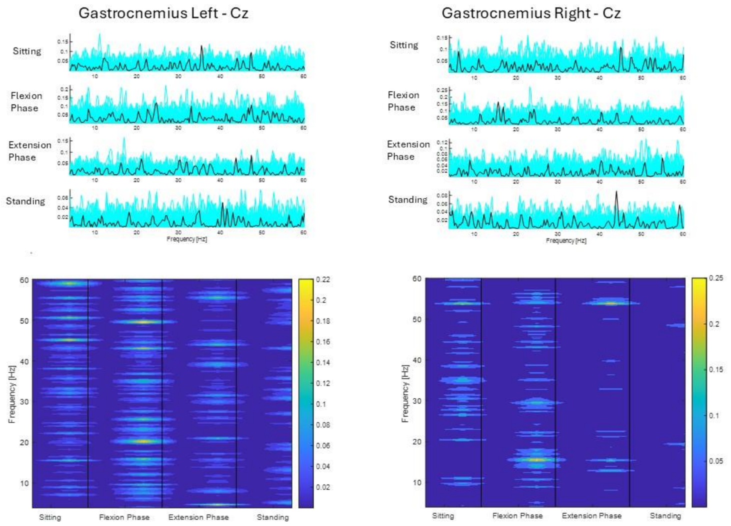

Figure S4

Subject 2: ERSP

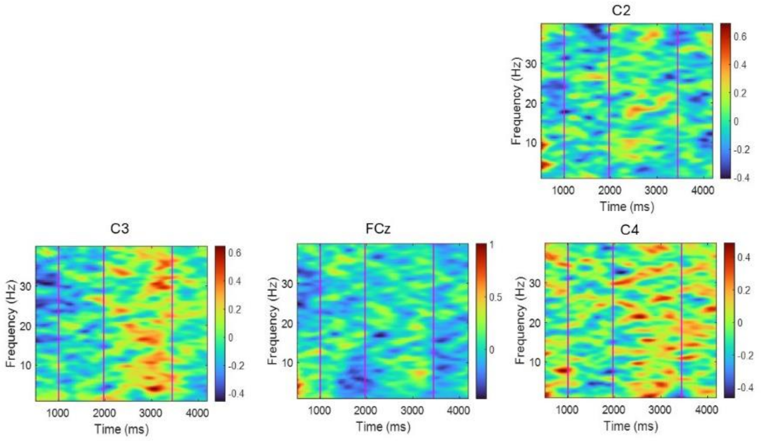

Figure S5

Subject 2: Topoplot

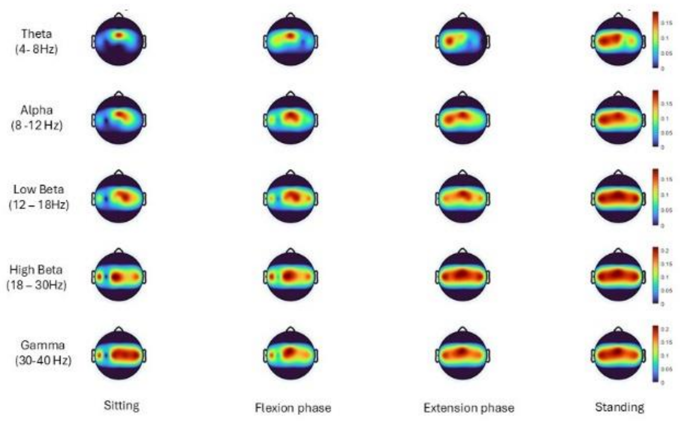

Figure S6

Subject 3: ERSP

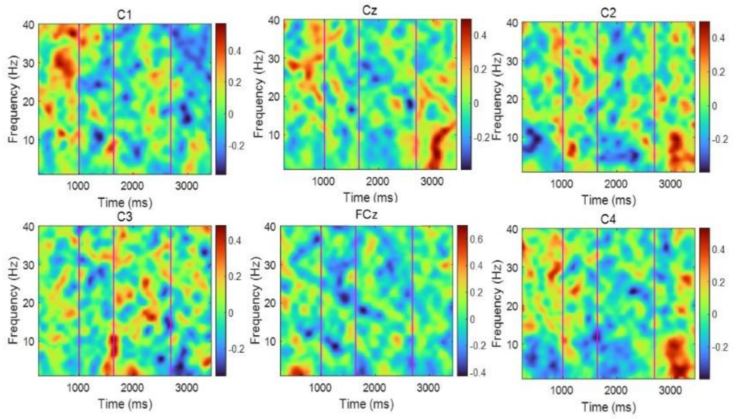

Figure S7

Subject 3: Topoplot

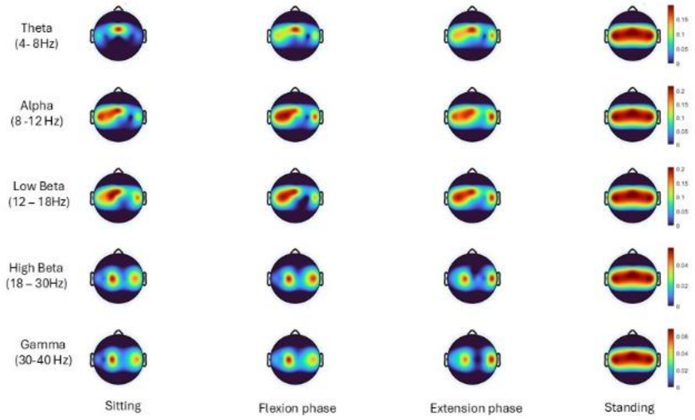

Figure S8

Subject 3: CMC

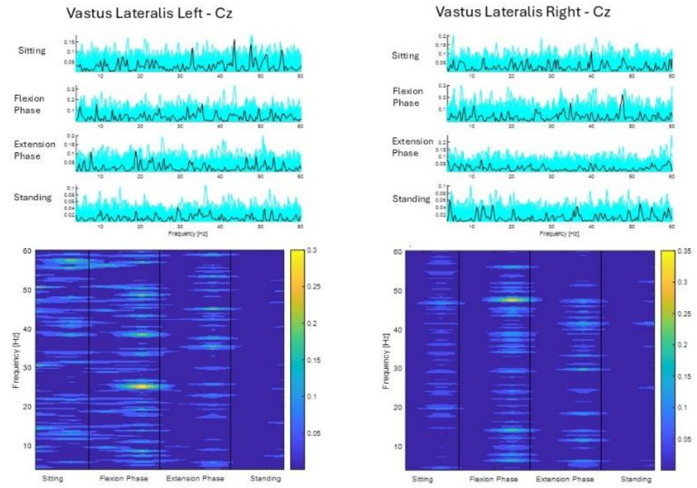

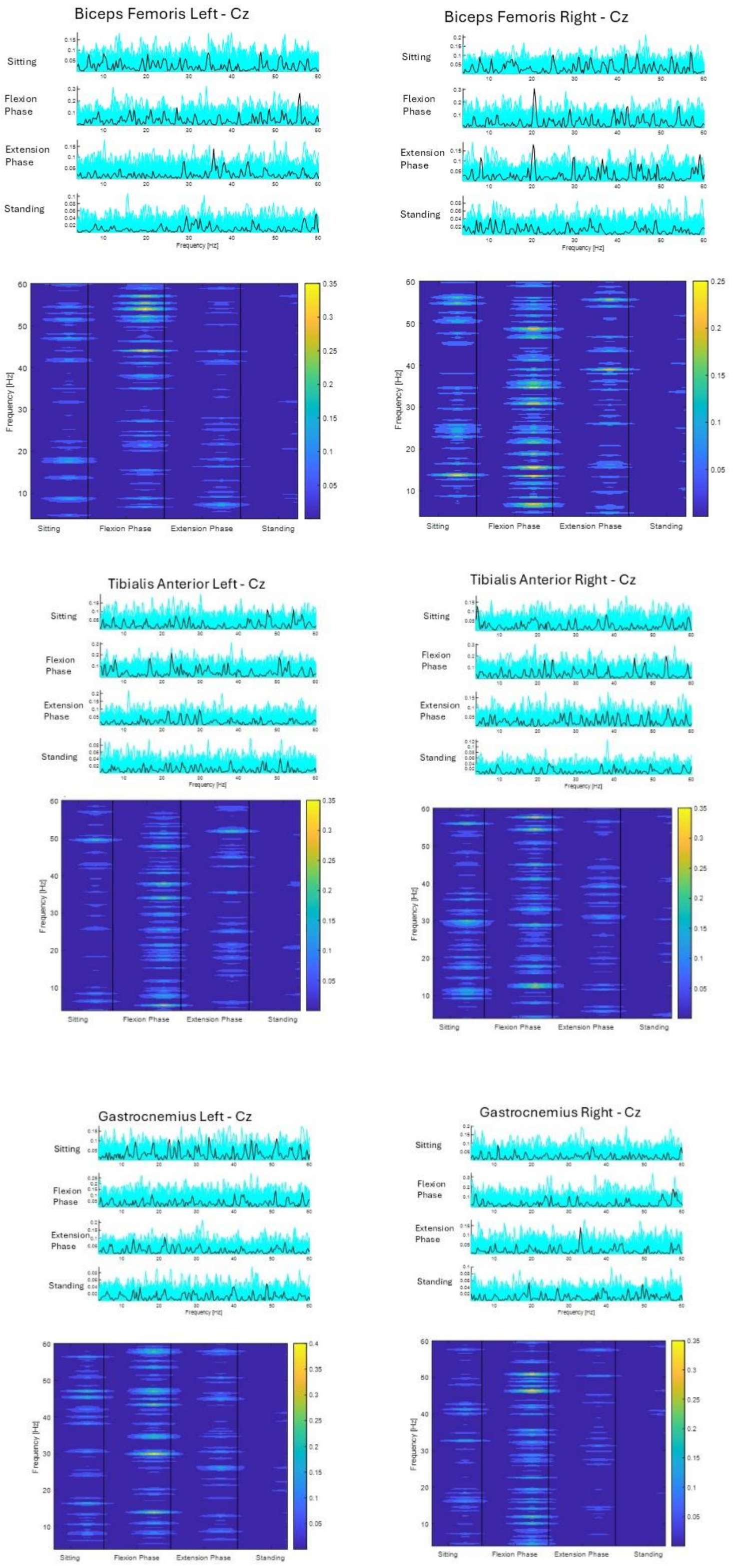

Figure S9

Subject 5: ERSP

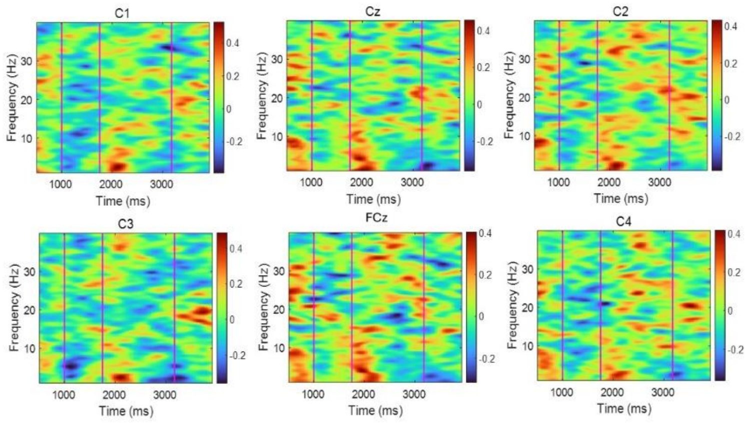

Figure S10

Subject 5: Topoplot

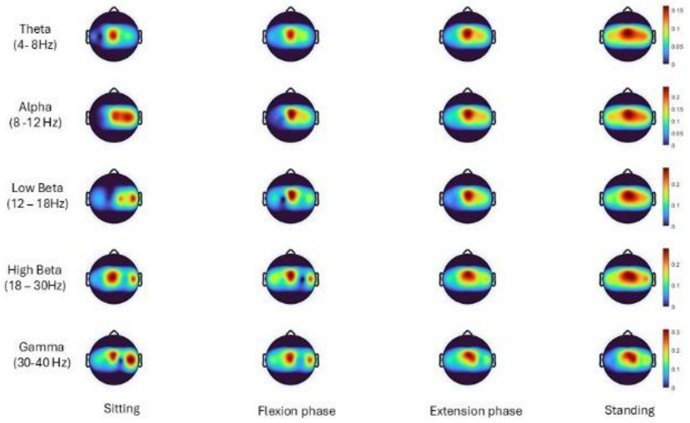

Figure S11

Subject 5: CMC

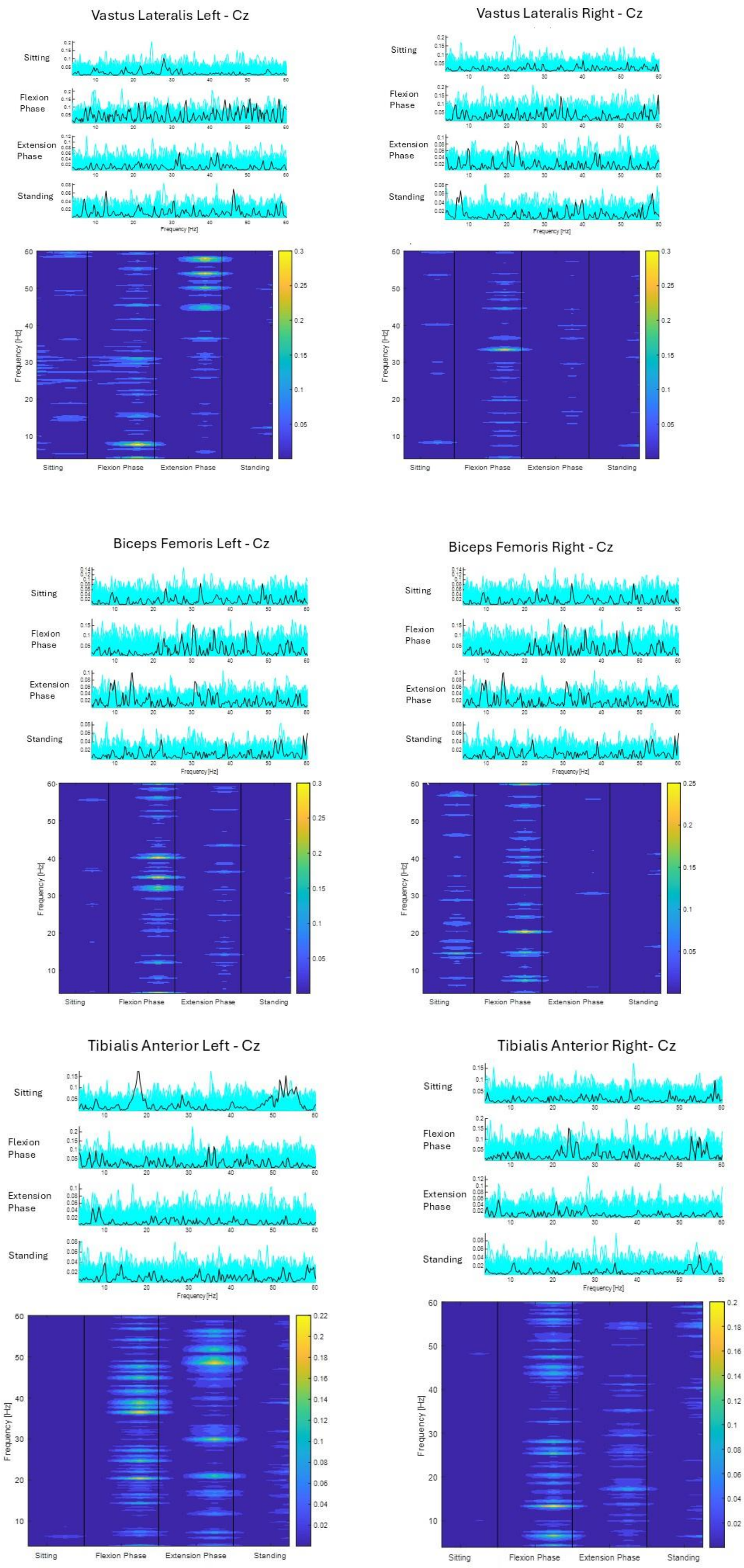
Vastus Lateralis Left - Cz
Vastus Lateralis Right - Cz
Biceps Femoris Left - Cz
Biceps Femoris Right - Cz
Tibialis Anterior Left - Cz
Tibialis Anterior Right- Cz
Sitting
Flexion Phase
Extension Phase
Standing
Frequency [Hz]

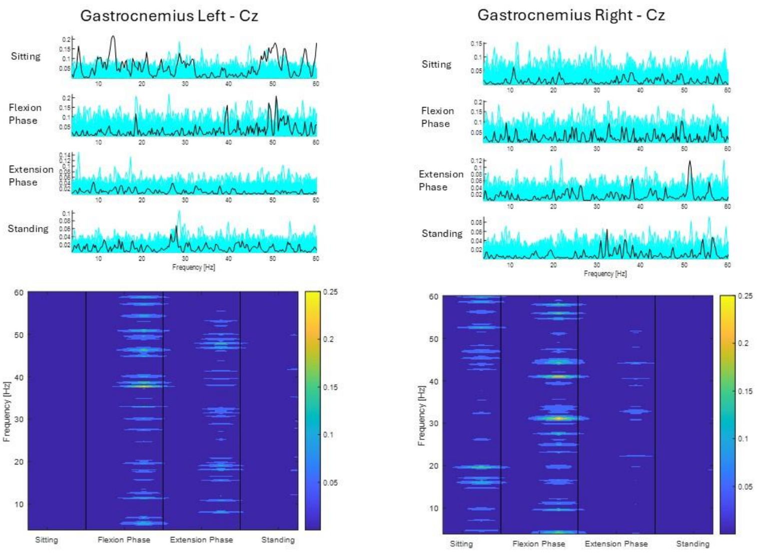

Figure S12

Subject 6: ERSP

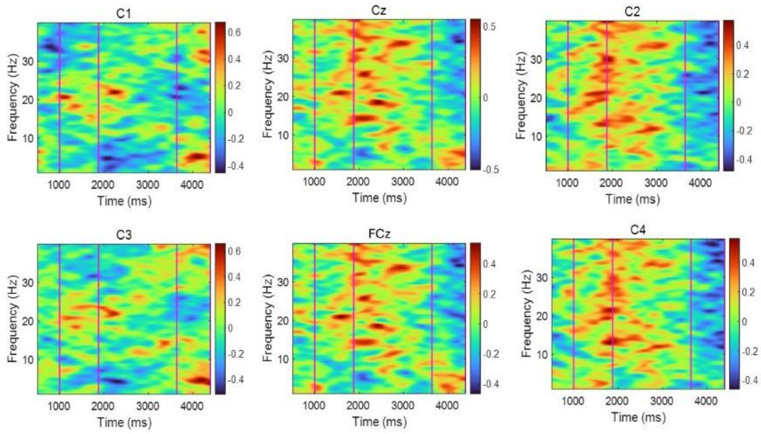

Figure S13

Subject 6: Topoplot

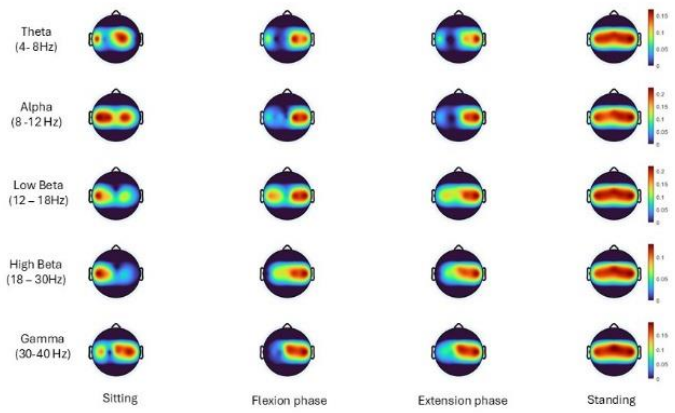

Figure S14

Subject 7: ERSP

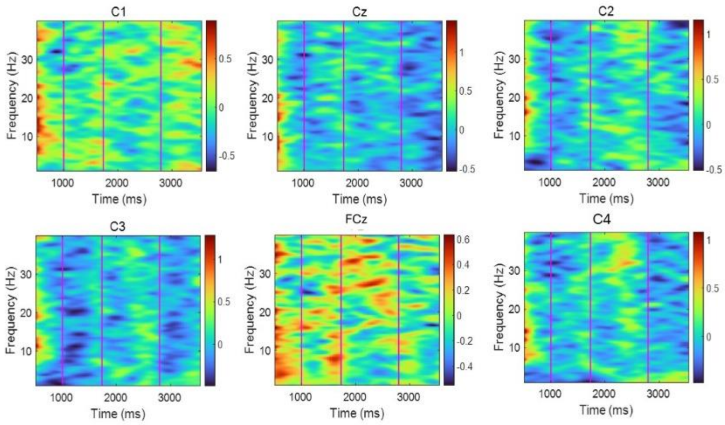

Figure S15

Subject 7: Topoplot

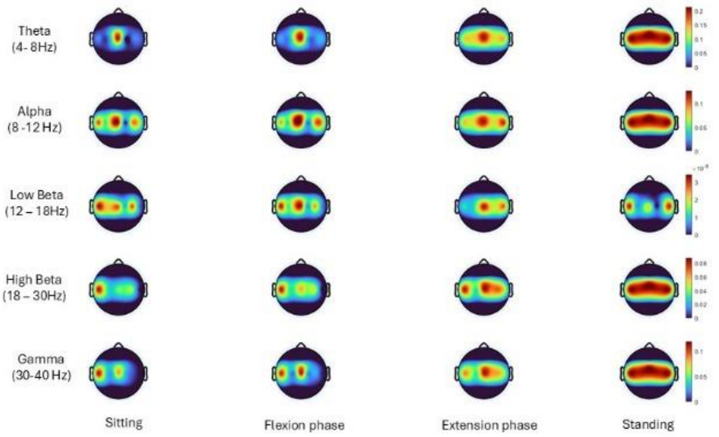

Figure S16

Subject 7: CMC

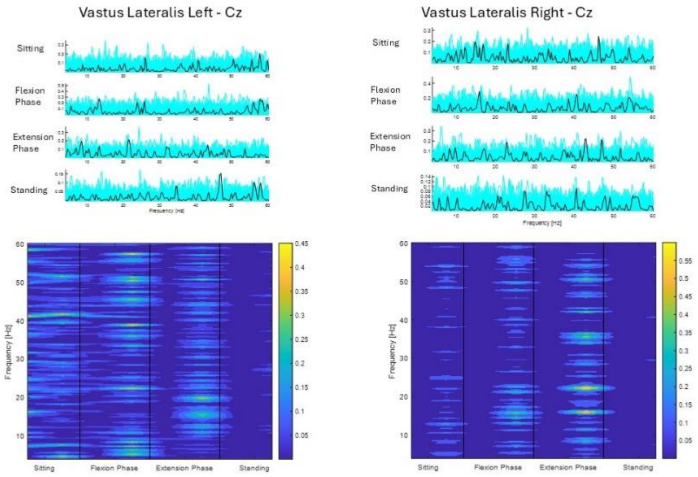

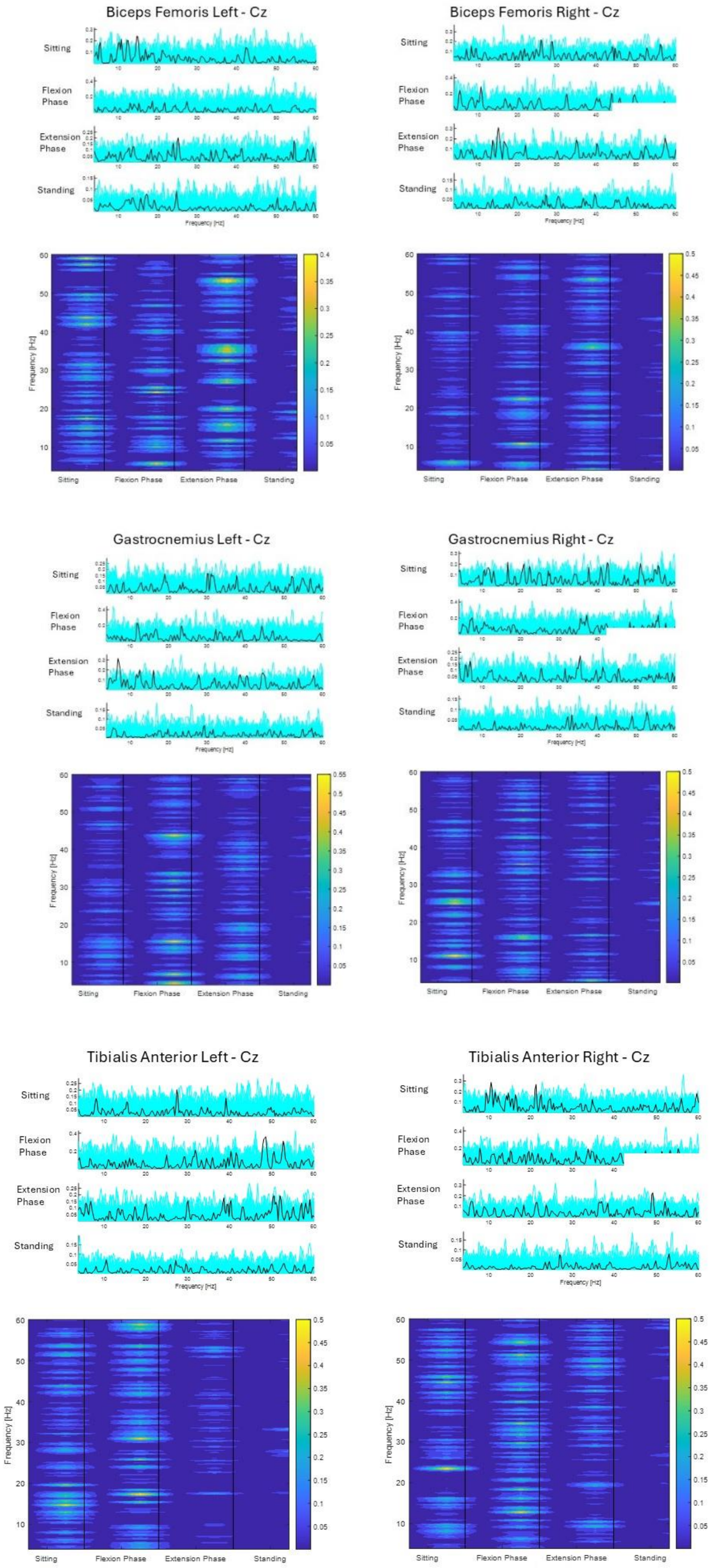

Figure S17

Subject 8: ERSP

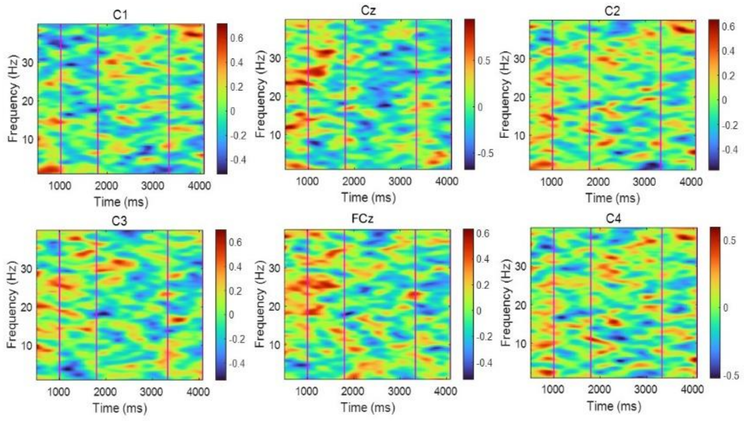

Figure S18

Subject 8: Topoplot

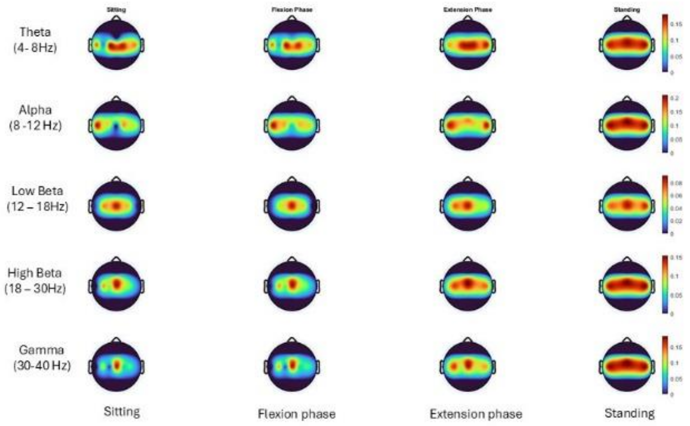

Figure S19

Subject 8: CMC

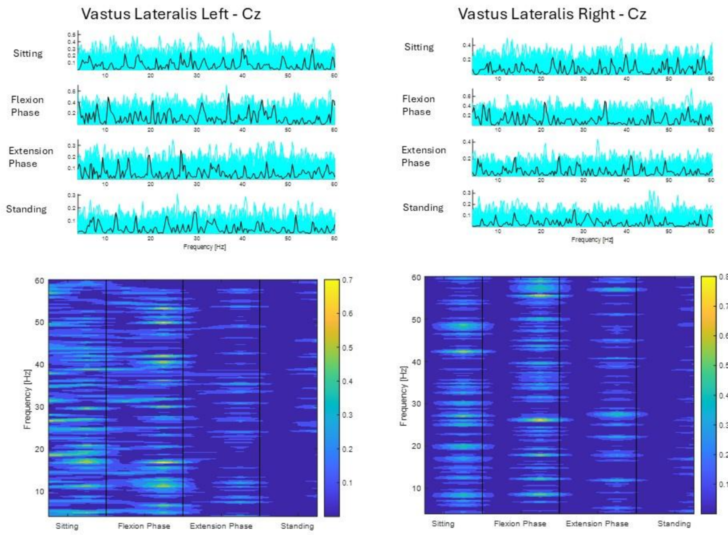

Vastus Lateralis Left - Cz
Vastus Lateralis Right - Cz
Sitting
Flexion Phase
Extension Phase
Standing
Frequency [Hz]

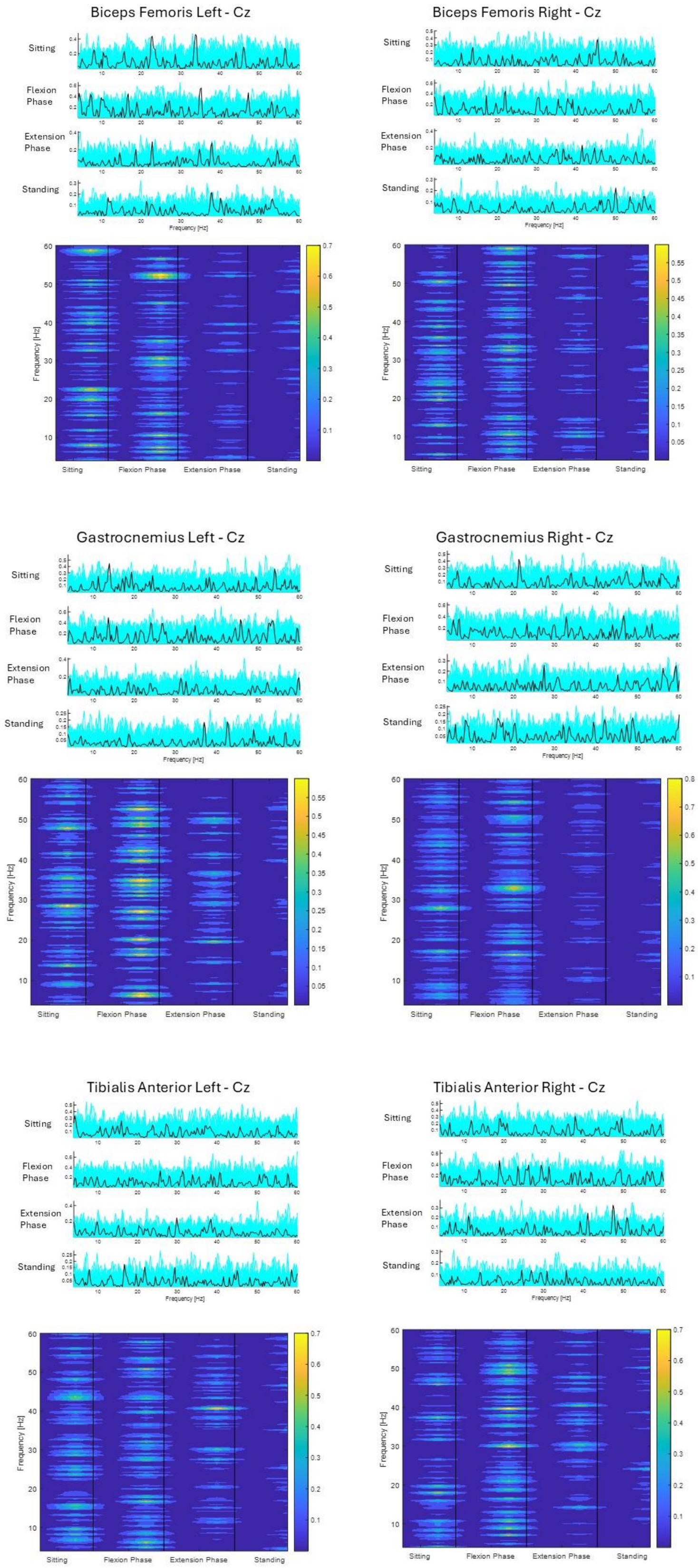
Biceps Femoris Left - Cz
Biceps Femoris Right - Cz
Gastrocnemius Left - Cz
Gastrocnemius Right - Cz
Tibialis Anterior Left - Cz
Tibialis Anterior Right - Cz
Sitting
Flexion Phase
Extension Phase
Standing
Frequency [Hz]

Figure S20

Subject 10: ERSP

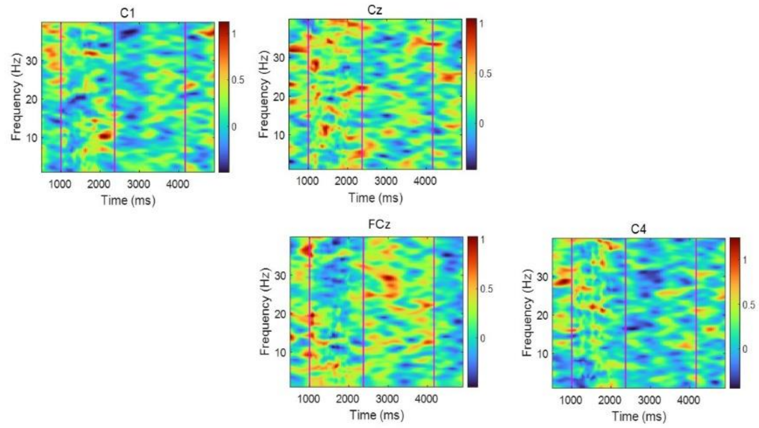

Figure S21

Subject 10: Topoplot

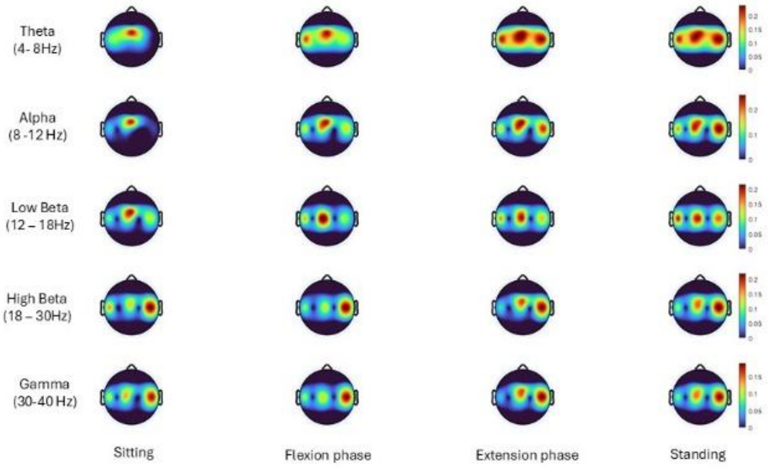

Figure S22

Subject 10: CMC

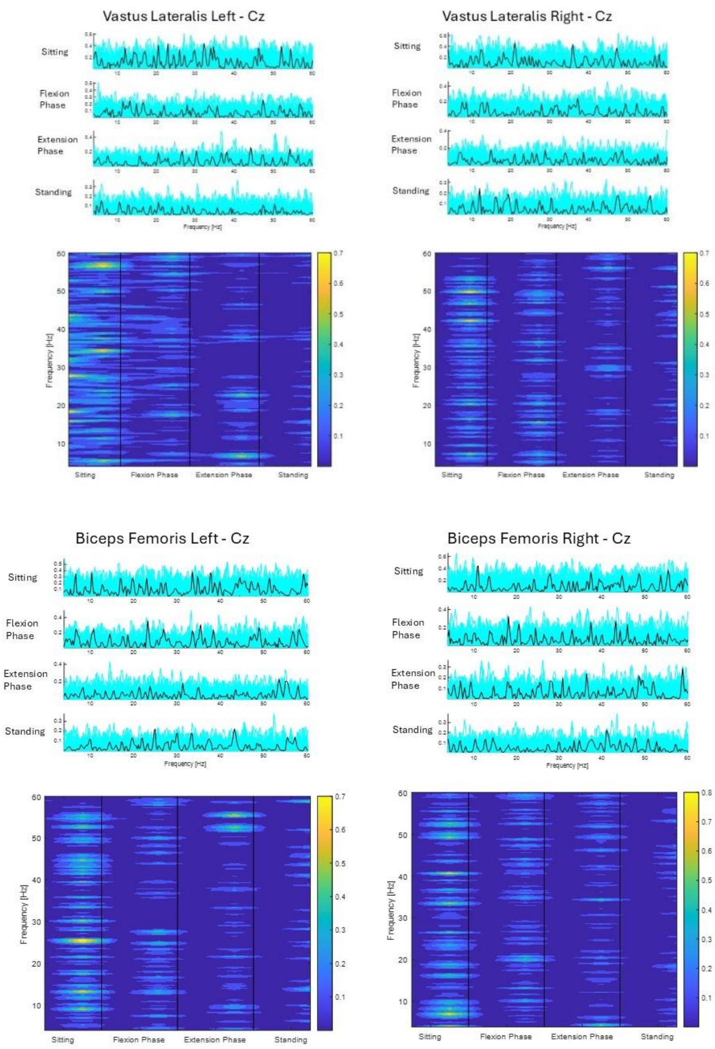

Vastus Lateralis Left - Cz
Vastus Lateralis Right - Cz
Sitting
Flexion Phase
Extension Phase
Standing
Frequency [Hz]
Biceps Femoris Left - Cz
Biceps Femoris Right - Cz

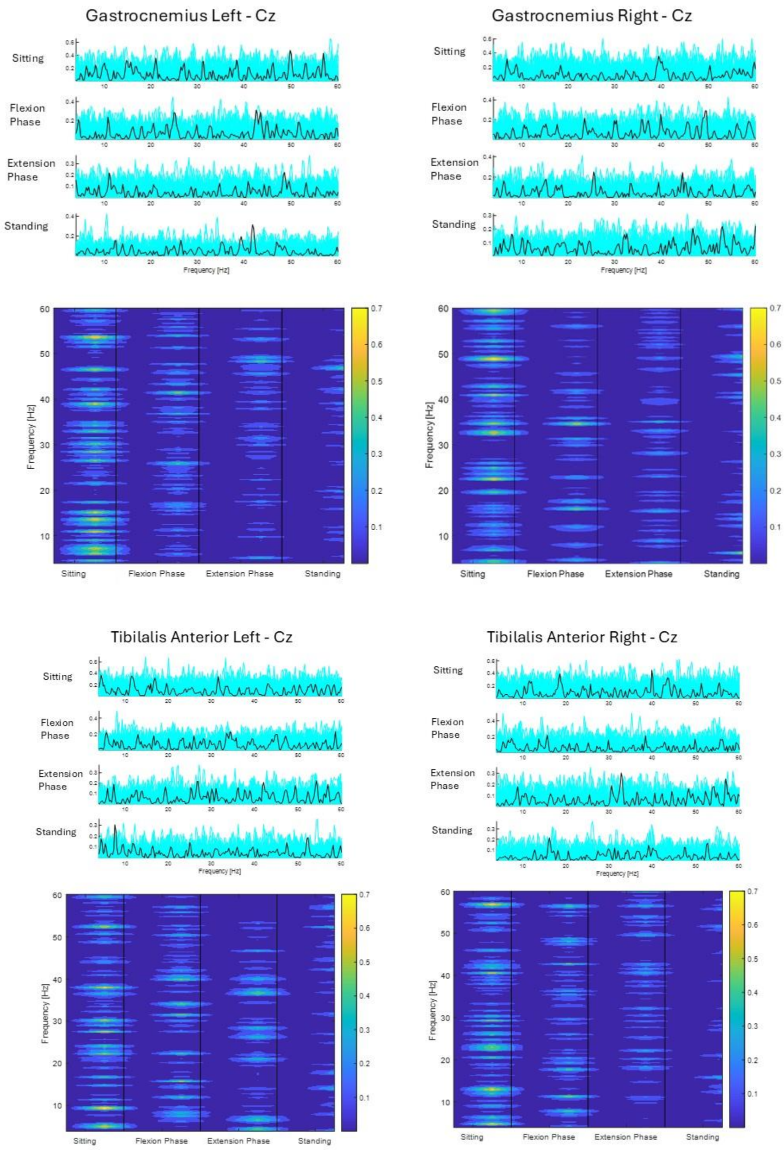

Figure S23

Subject 11: ERSP

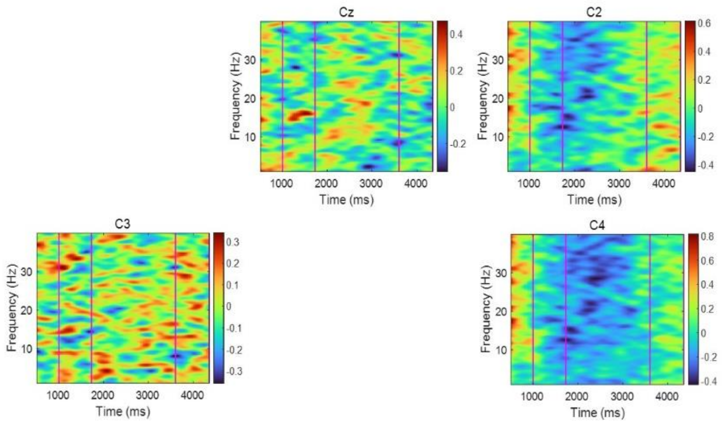

Figure S24

Subject 11: Topoplot

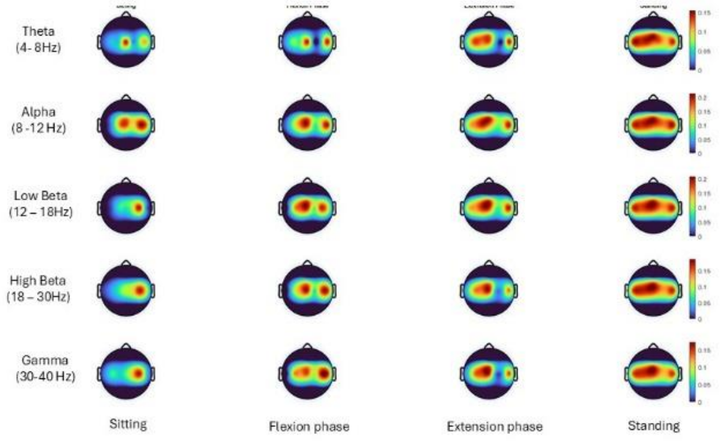

Figure S25

Subject 11: CMC

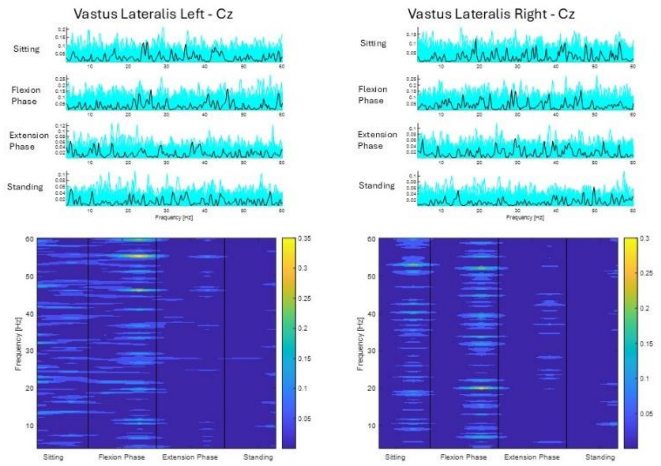

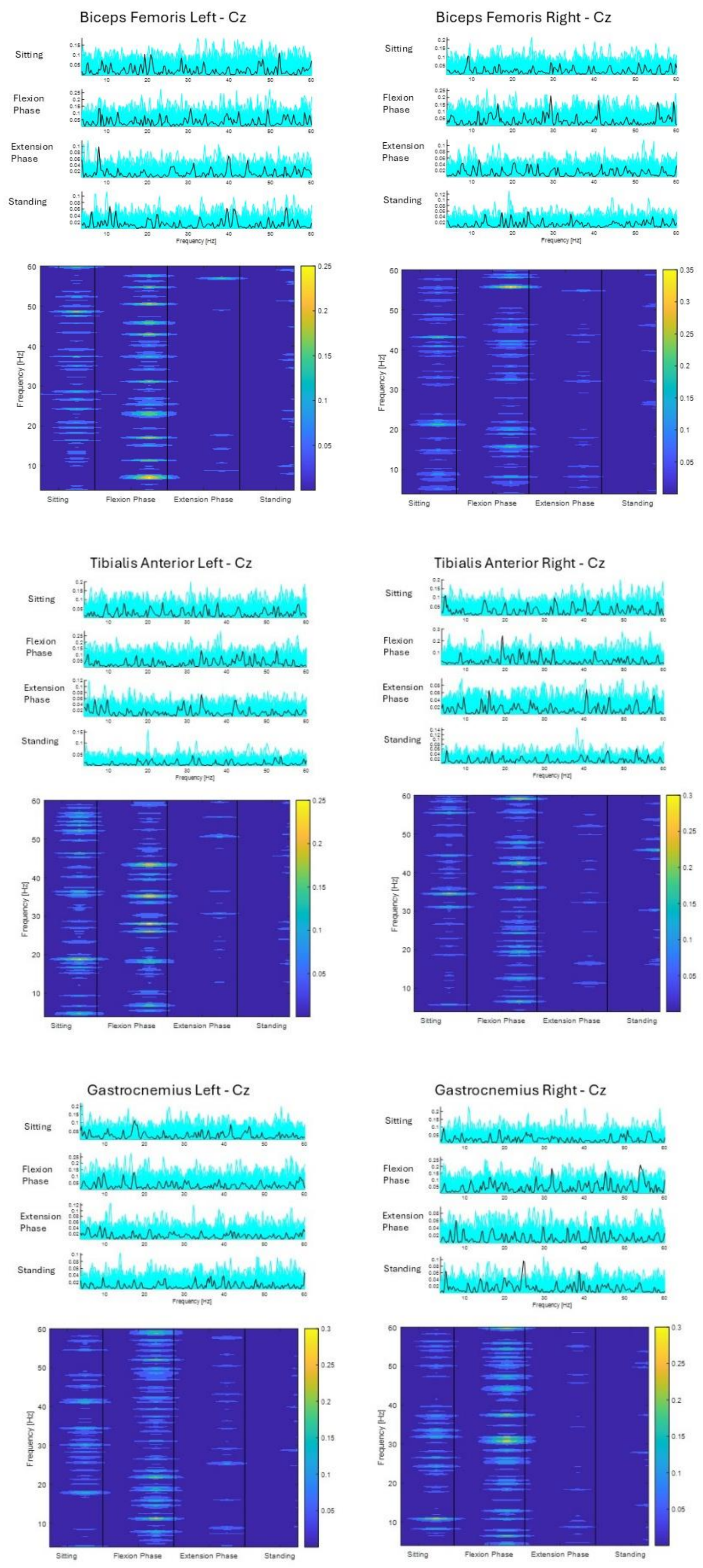

Figure S26

Subject 12: ERSP

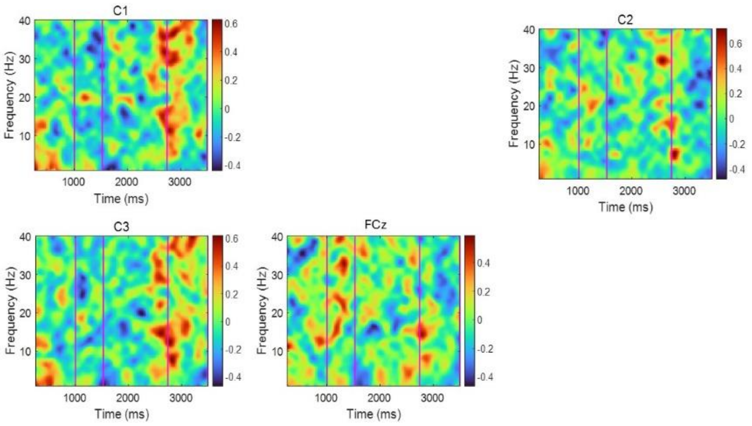

Figure S27

Subject 12: Topoplot

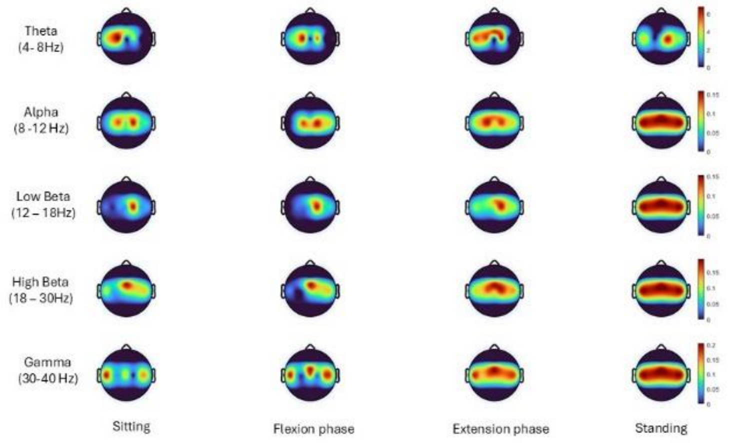

Figure S28

Subject 13: ERSP

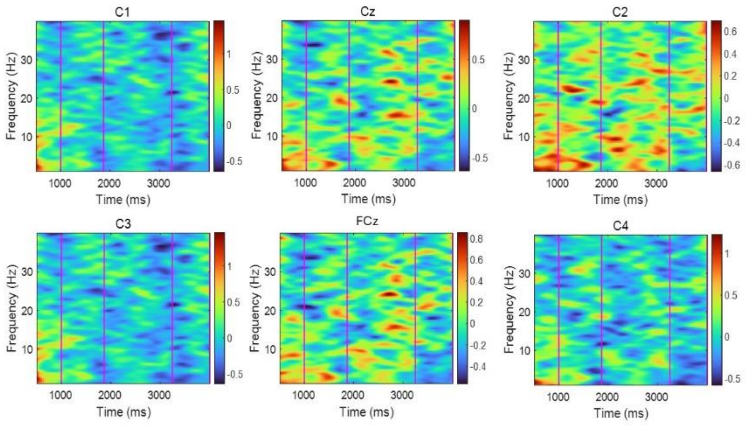

Figure S29

Subject 13: Topoplot

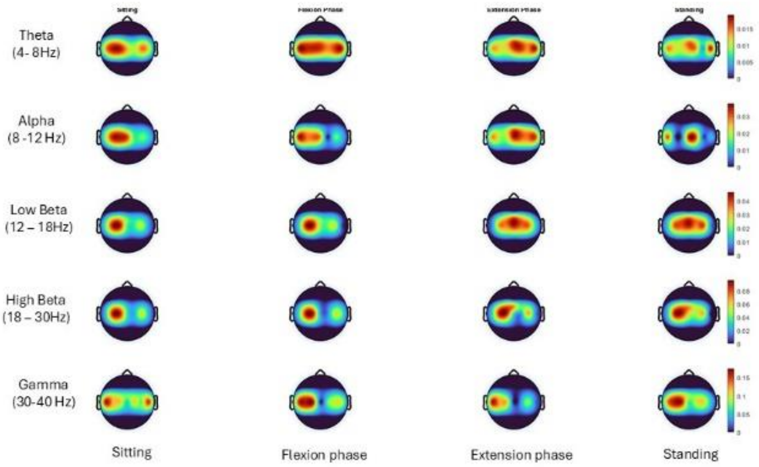

Figure S30

Subject 13: CMC

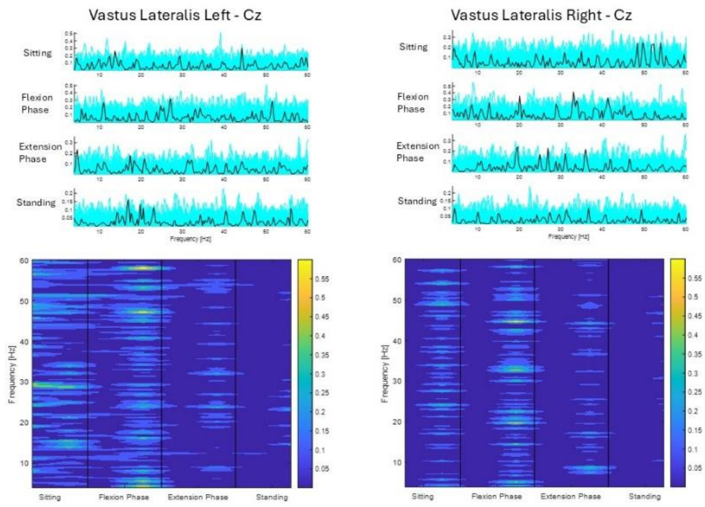

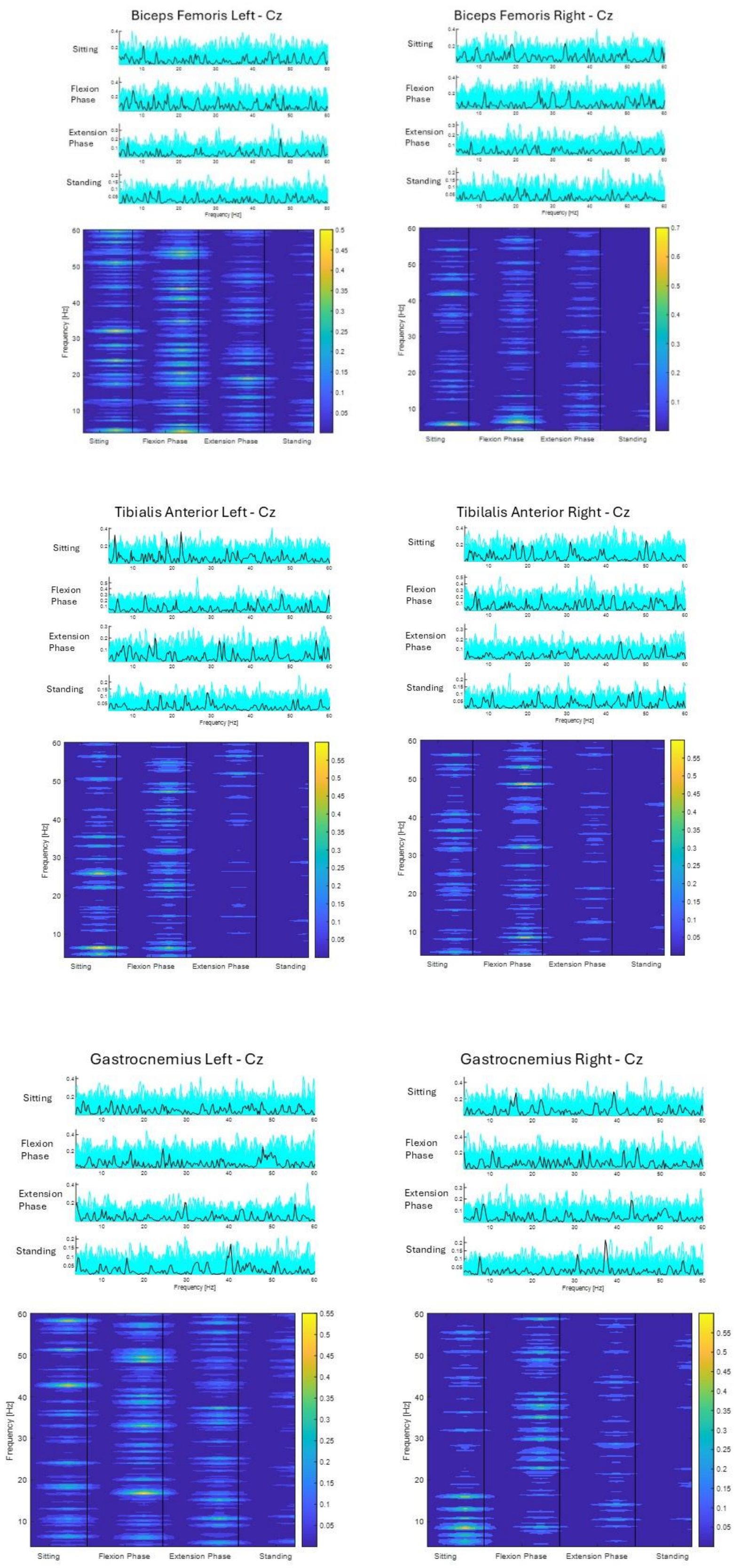

Figure S31

Subject 14: ERSP

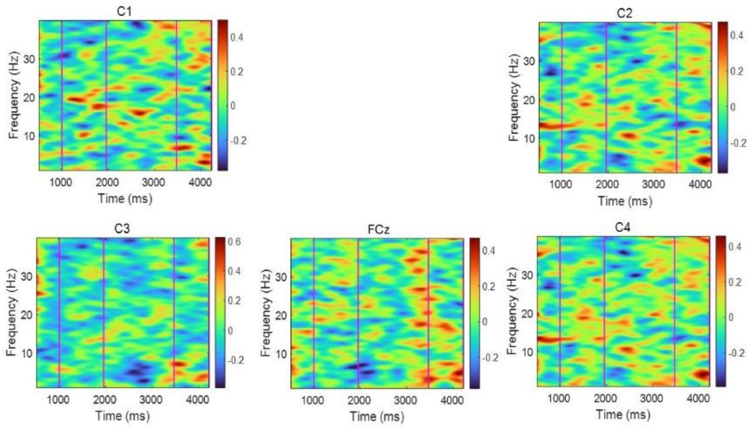

Figure S32

Subject 14: Topoplot

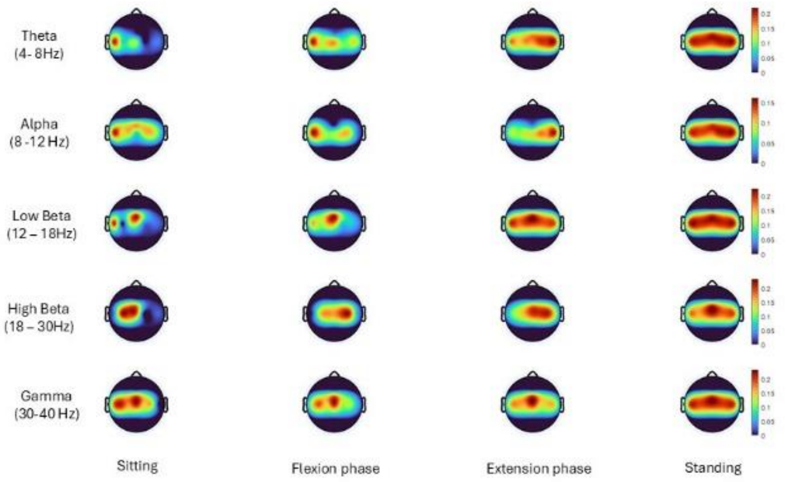

Figure S33

Subject 15: ERSP

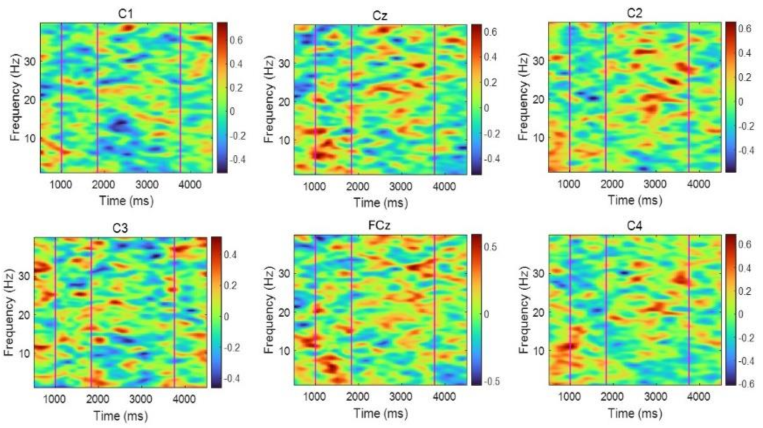

Figure S34

Subject 15: Topoplot

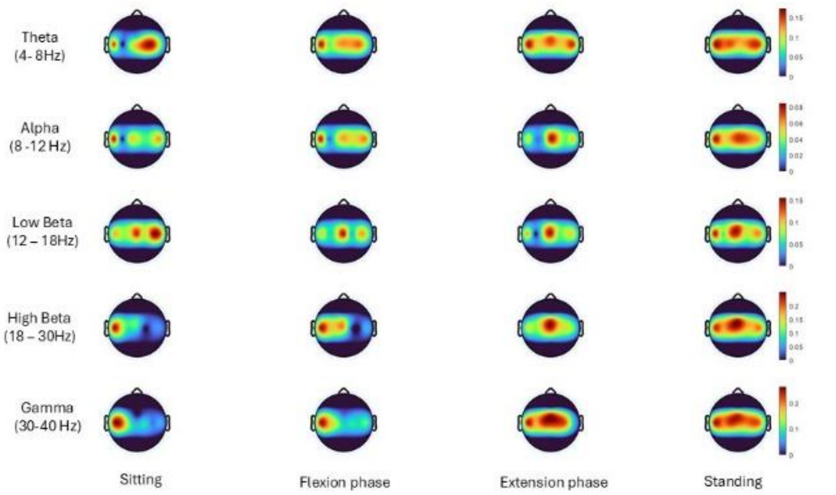

Figure S35

Subject 15: CMC

Vastus Lateralis Left - Cz

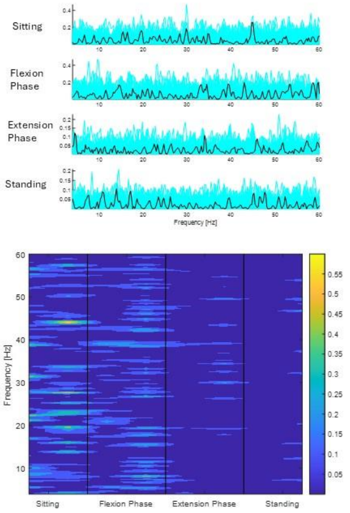

Vastus Lateralis Right - Cz

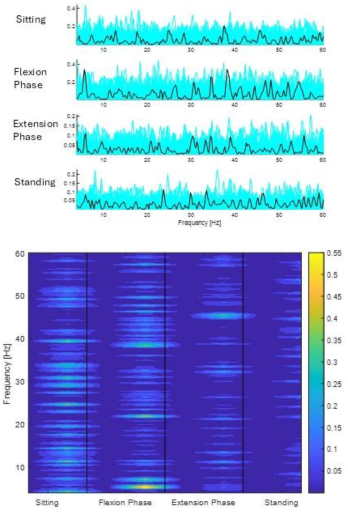

Biceps Femoris Left - Cz

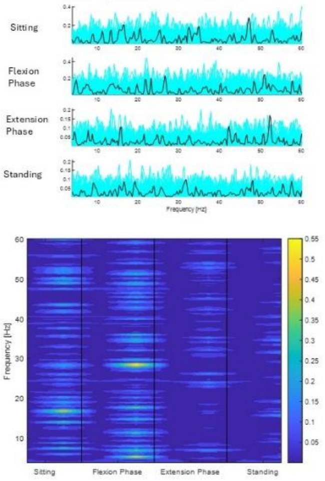

Biceps Femoris Right - Cz

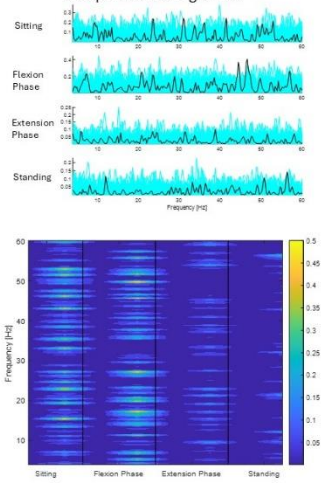

Tibialis Anterior Left - Cz

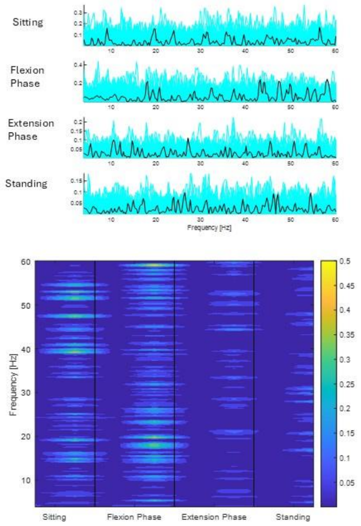

Tibialis Anterior Right - Cz

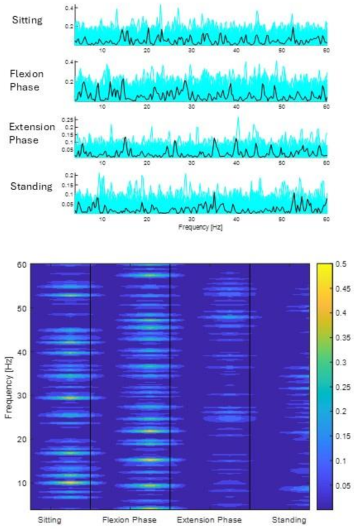

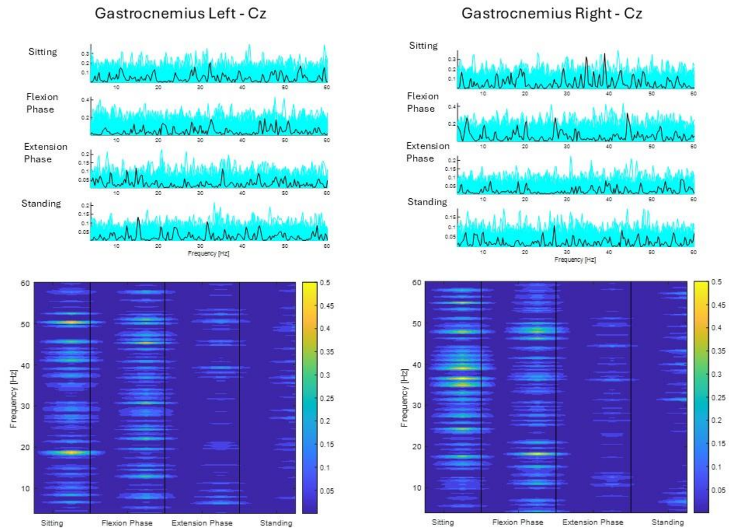

Figure S36

Subject 17: ERSP

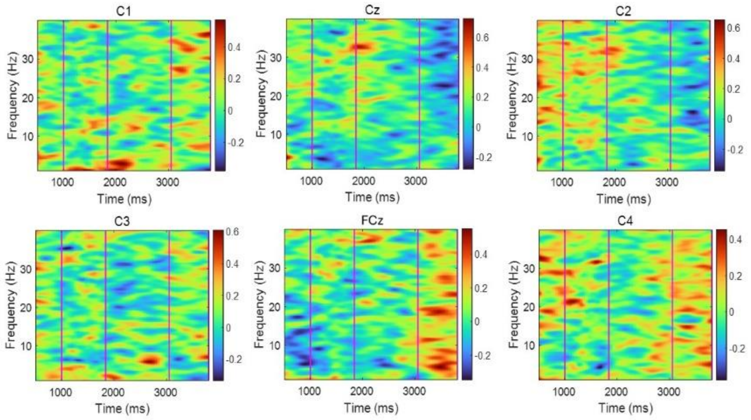

Figure S37

Subject 17: Topoplot

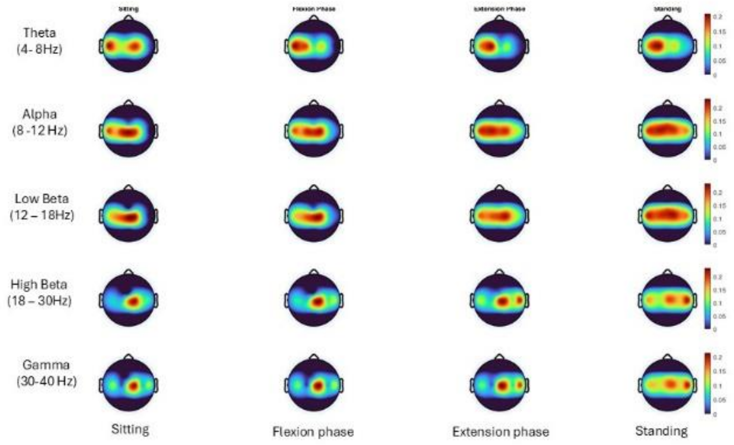

Figure S38

Subject 17: CMC

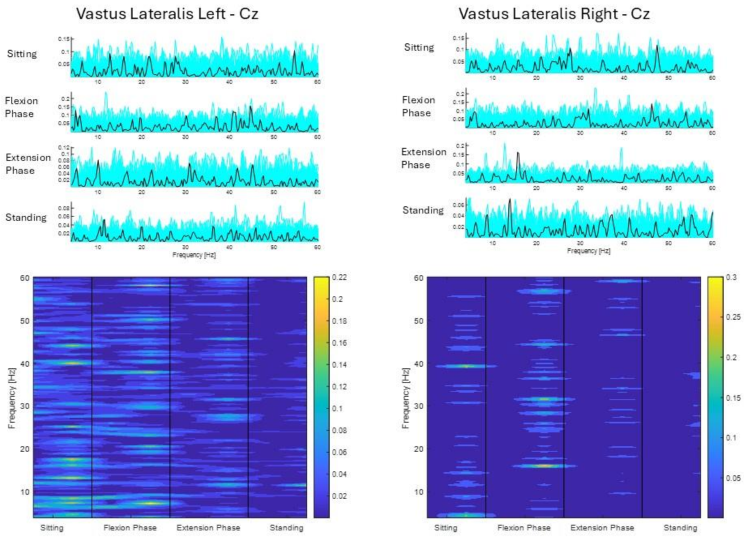

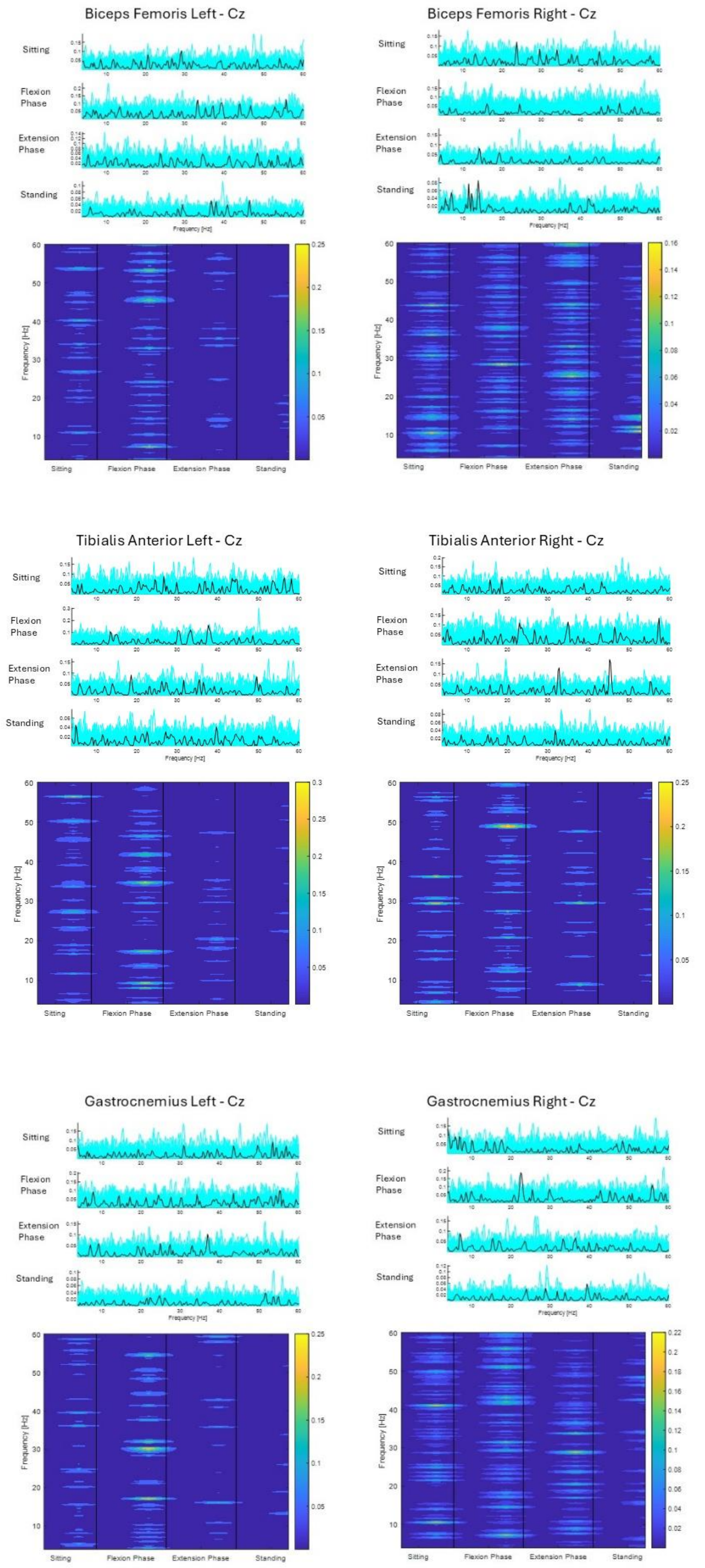

Figure S39

Subject 18: ERSP

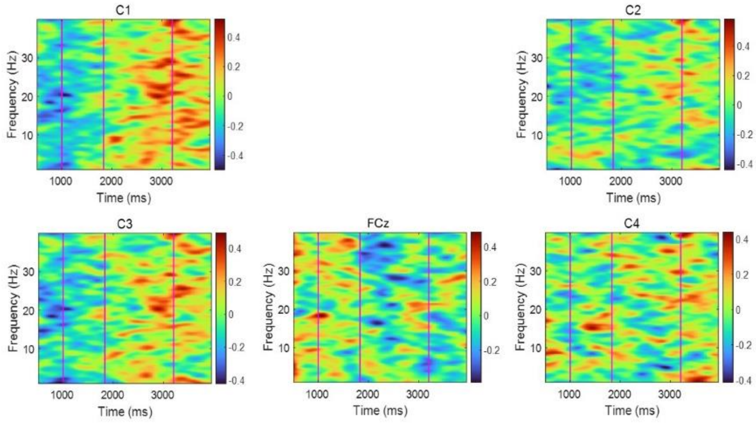

Figure S40

Subject 18: Topoplot

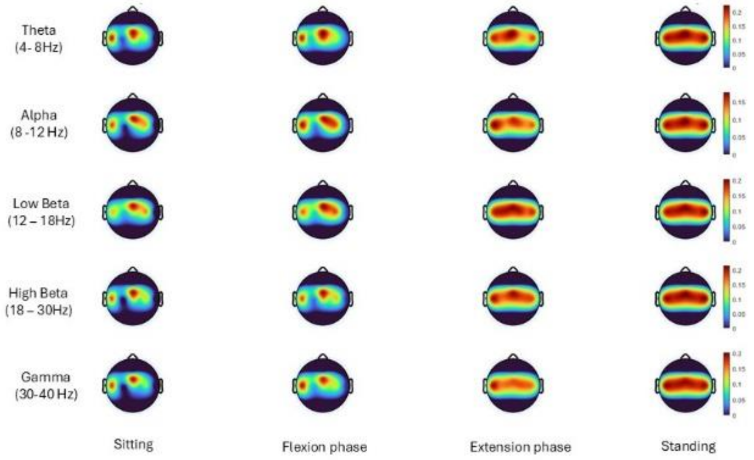

Figure S41

Subject 18: CMC

Vastus Lateralis Left – Cz

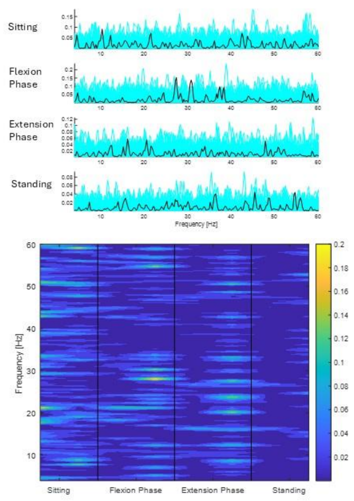

Vastus Lateralis Right – Cz

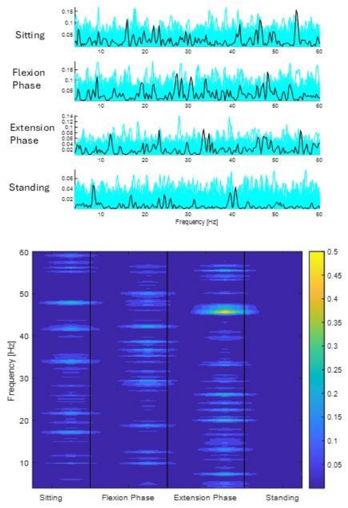

Biceps Femoris Left – Cz

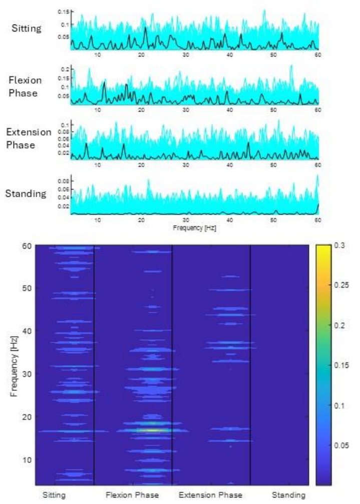

Biceps Femoris Right – Cz

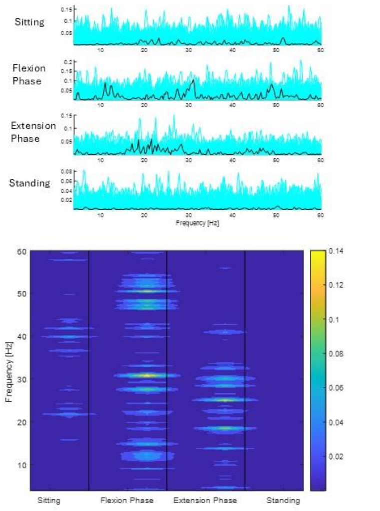

Tibialis Anterior Left – Cz

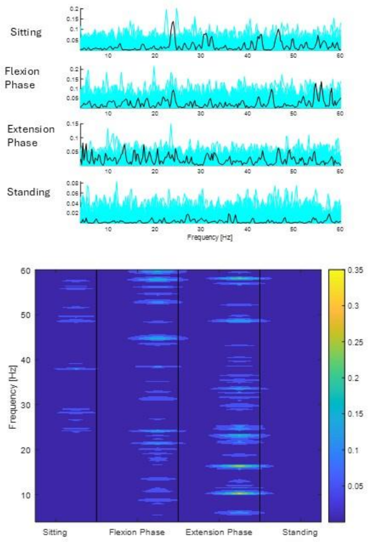

Tibialis Anterior Right – Cz

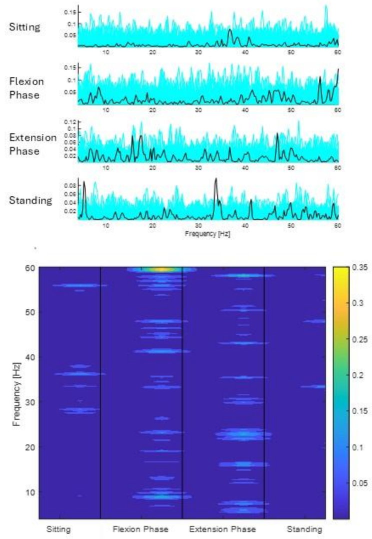

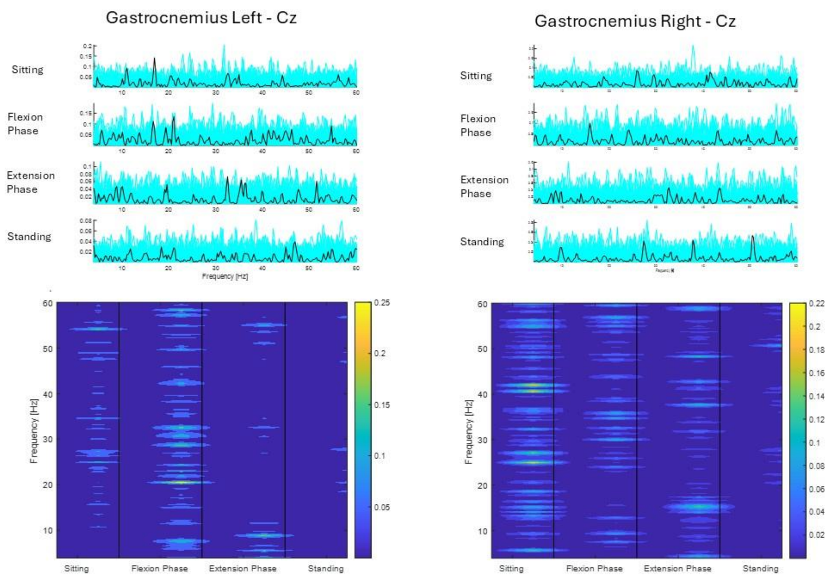

Figure S42

Subject 19: ERSP

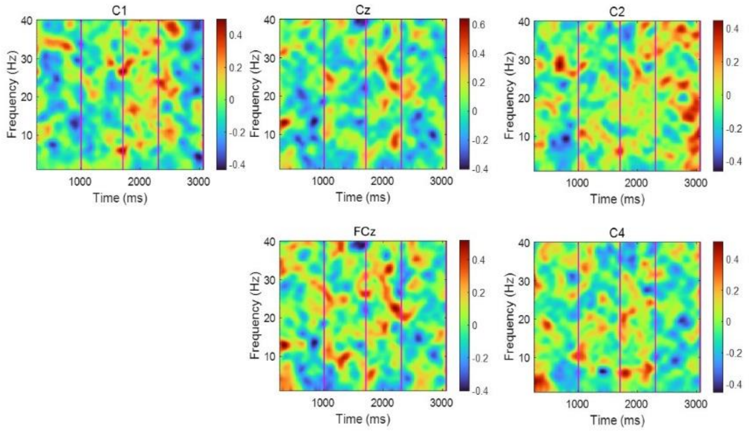

Figure S43

Subject 19: Topoplot

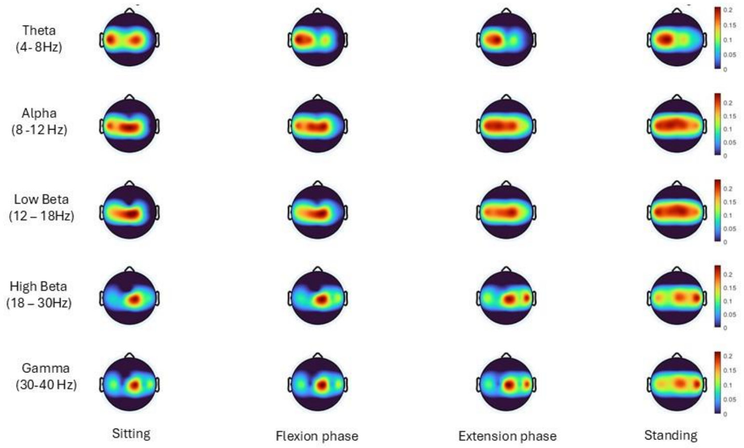

Figure S44

Subject 19: CMC

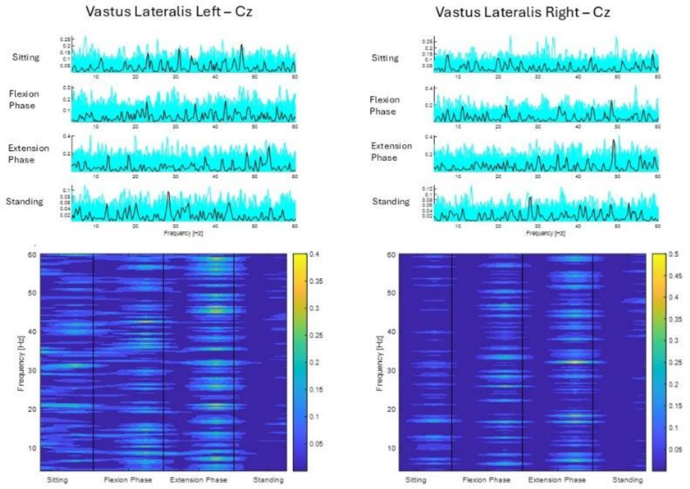

Biceps Femoris Left – Cz

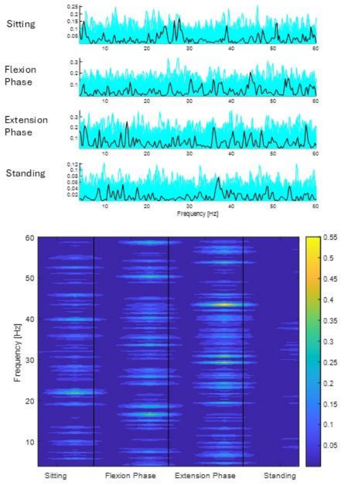

Biceps Femoris Right – Cz

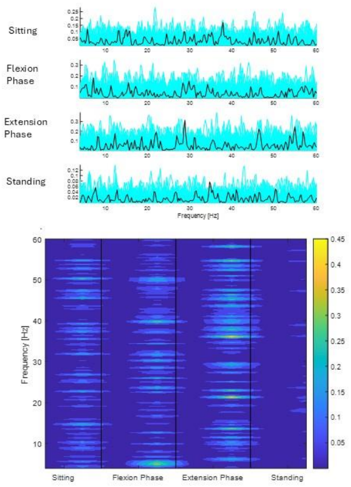

Tibialis Anterior Left – Cz

No viable EMG data

Tibialis Anterior Right - Cz

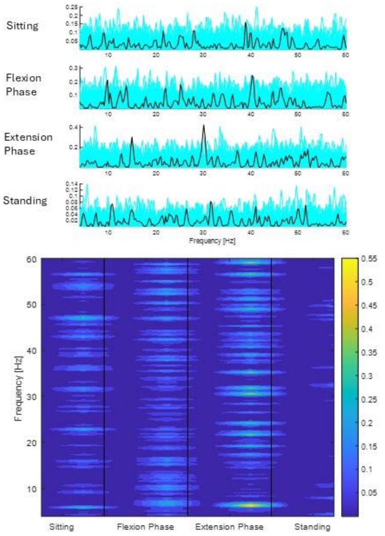

Gastrocnemius Left - Cz

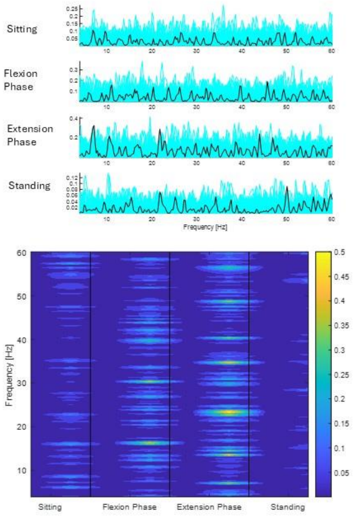

Gastrocnemius Right - Cz

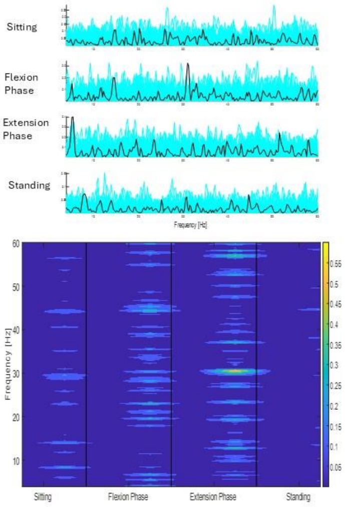

Figure S45

Subject 20: ERSP

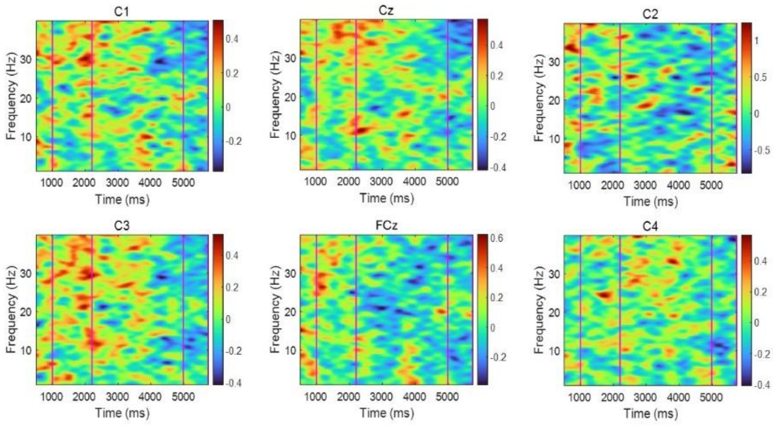

Figure S46

Subject 20: CMC

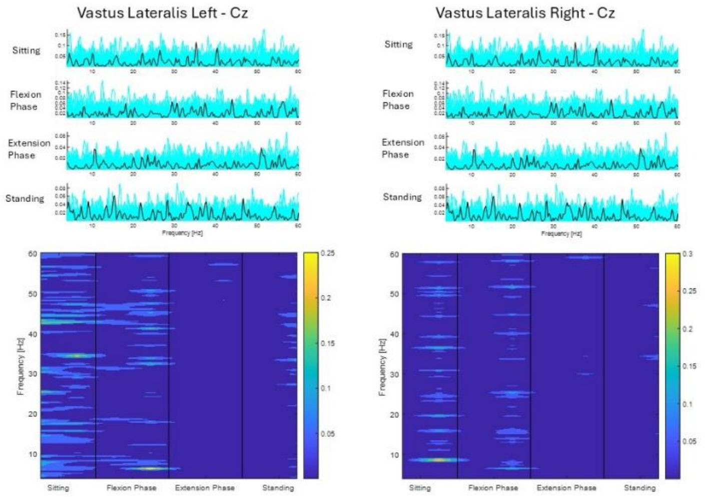

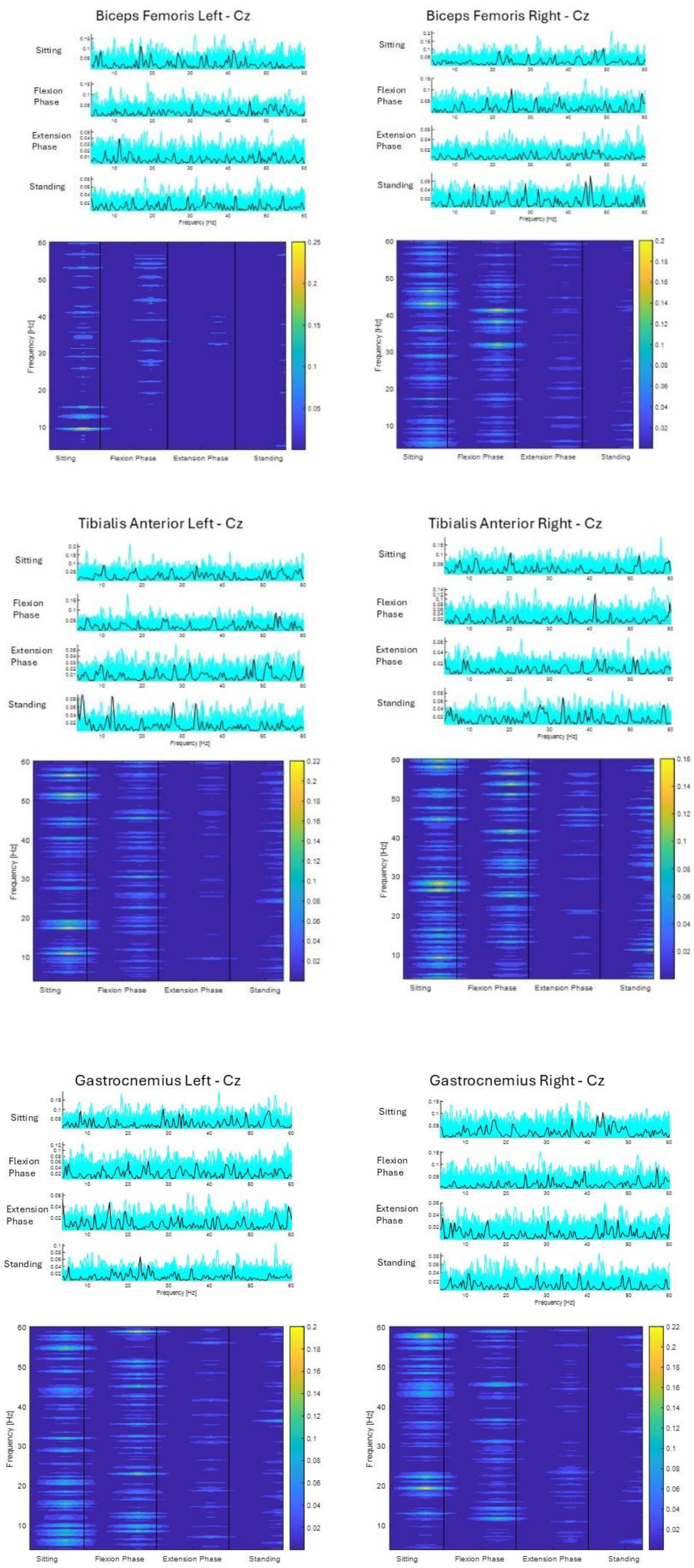

Figure S47

Subject 21: ERSP

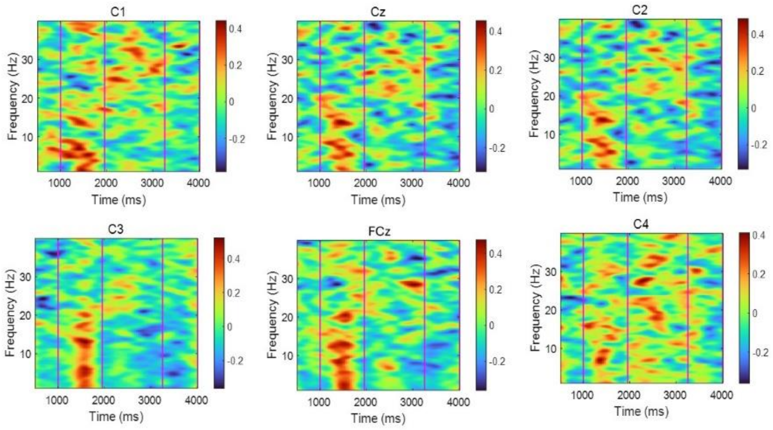

Figure S48

Subject 21: Topoplot

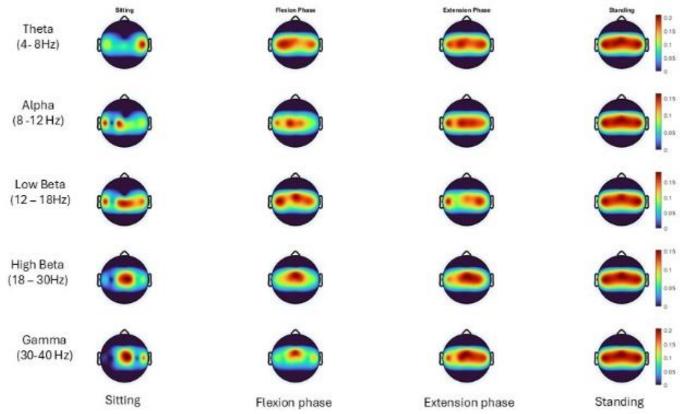

Figure S49

Subject 21: CMC

Vastus Lateralis Left - Cz

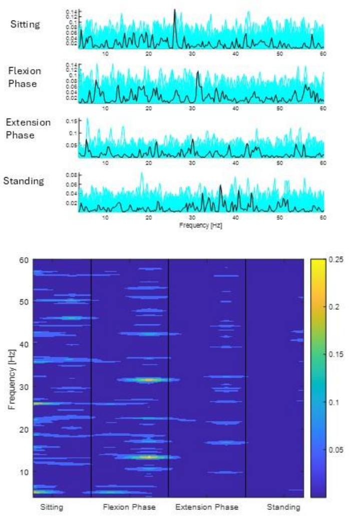

Vastus Lateralis Right - Cz

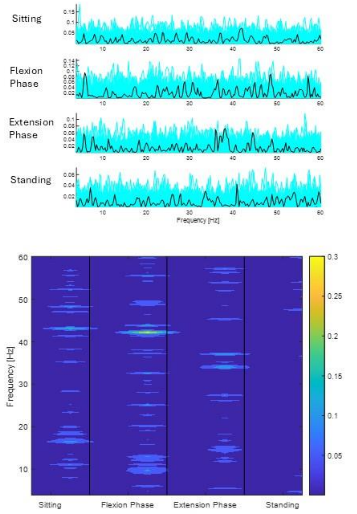

Biceps Femoris Left - Cz

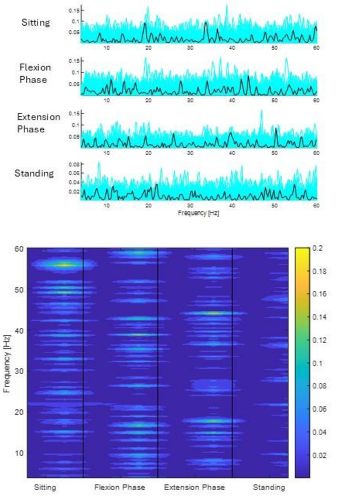

Biceps Femoris Right - Cz

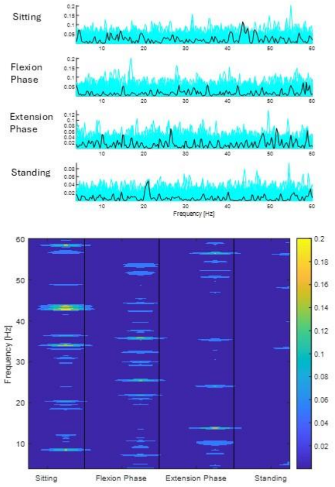

Tibialis Anterior Left - Cz

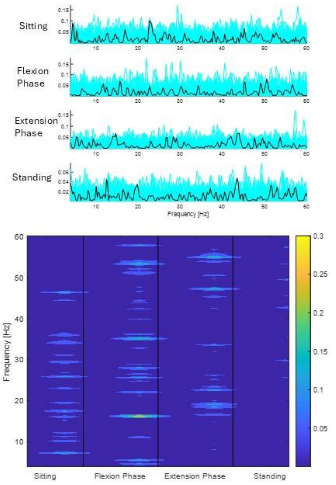

Tibialis Anterior Right - Cz

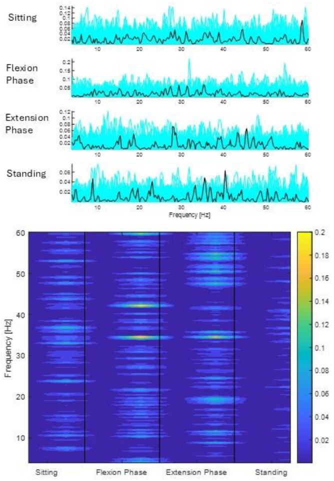

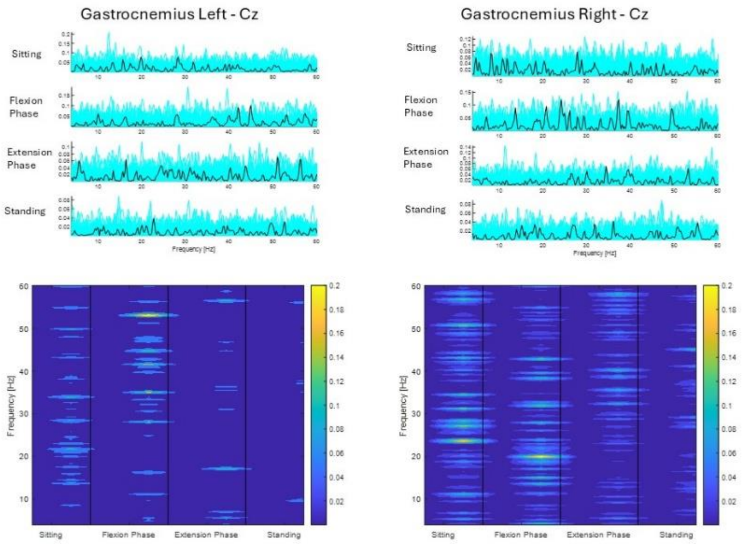

Figure S50

Subject 22: ERSP

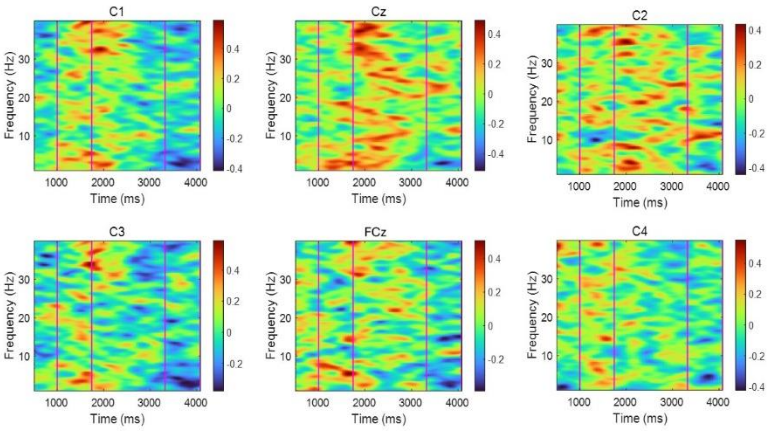

Figure S51

Subject 22: Topoplot

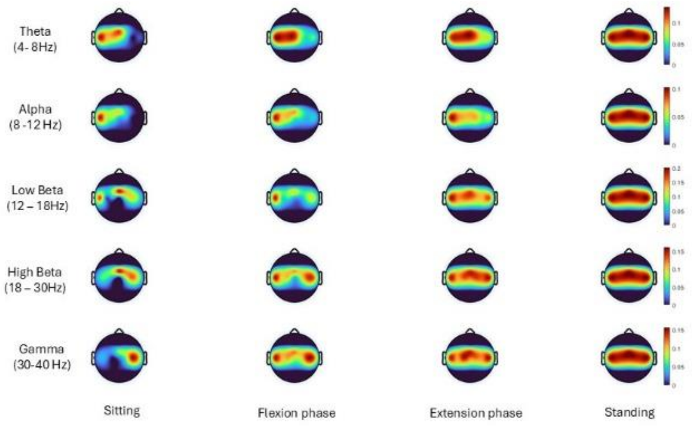

Figure S52

Subject 22: CMC

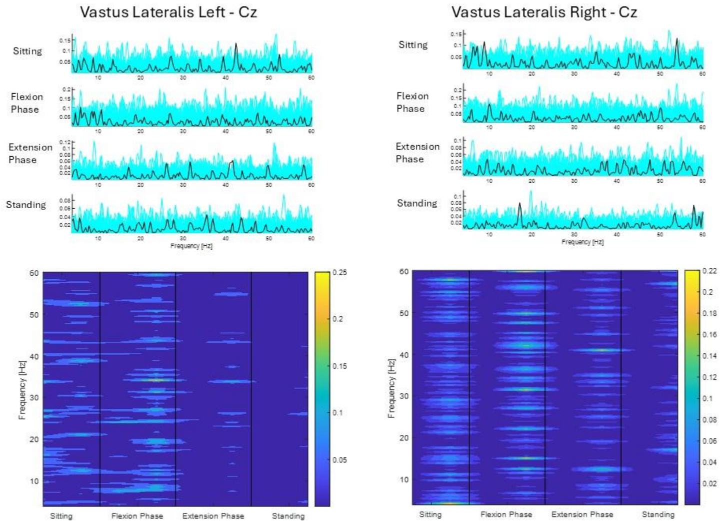

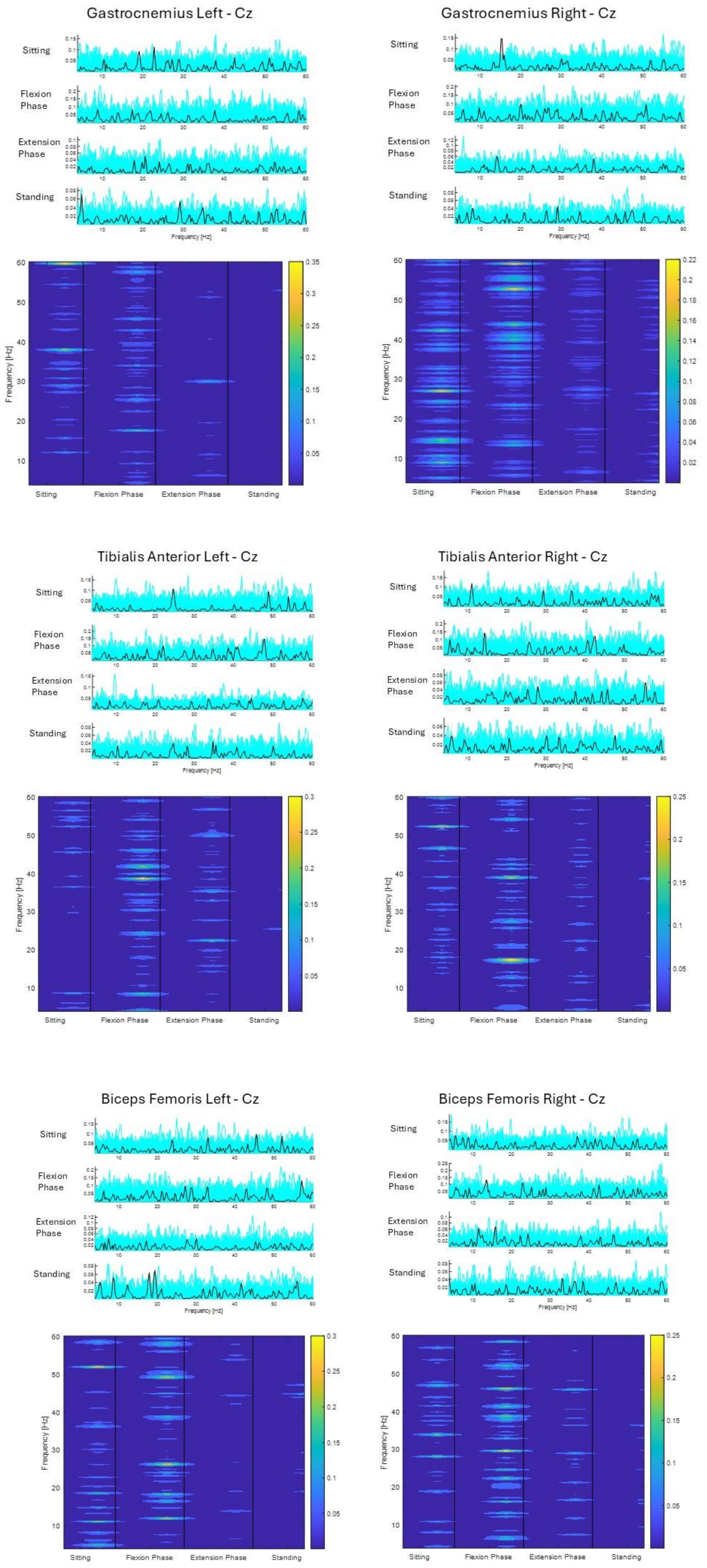

Figure S53

Subject 23: ERSP

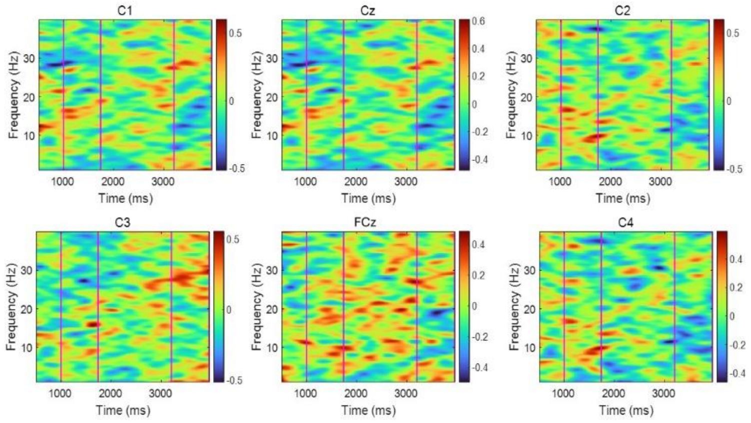

Figure S54

Subject 23: Topoplot

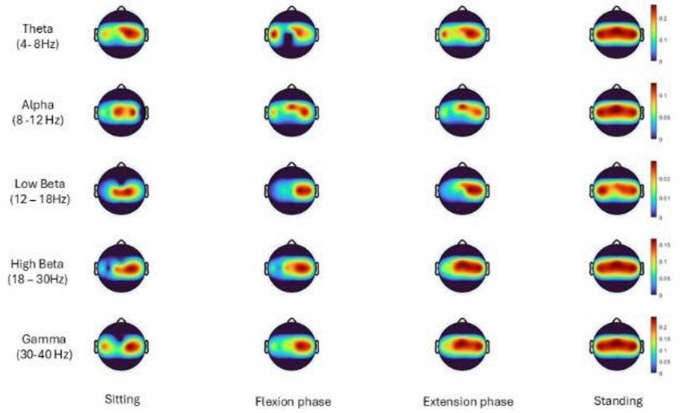

Figure S55

Subject 23: CMC

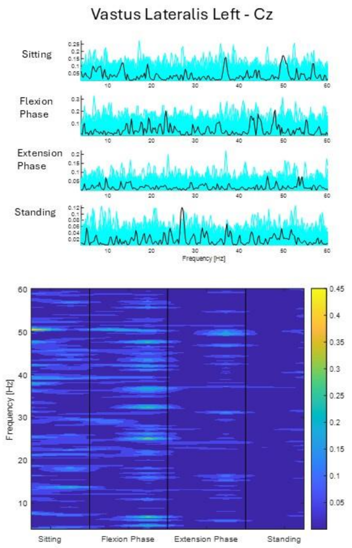
Vastus Lateralis Left - Cz
Sitting
Flexion Phase
Extension Phase
Standing
Frequency [Hz]
Frequency [Hz]
Sitting
Flexion Phase
Extension Phase
Standing

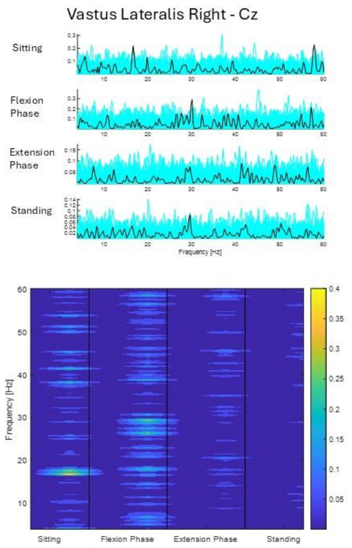
Vastus Lateralis Right - Cz
Sitting
Flexion Phase
Extension Phase
Standing
Frequency [Hz]
Frequency [Hz]
Sitting
Flexion Phase
Extension Phase
Standing

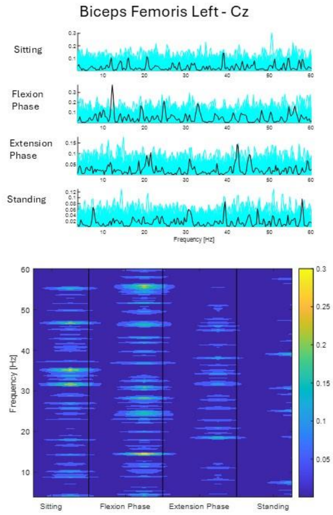
Biceps Femoris Left - Cz
Sitting
Flexion Phase
Extension Phase
Standing
Frequency [Hz]
Frequency [Hz]
Sitting
Flexion Phase
Extension Phase
Standing

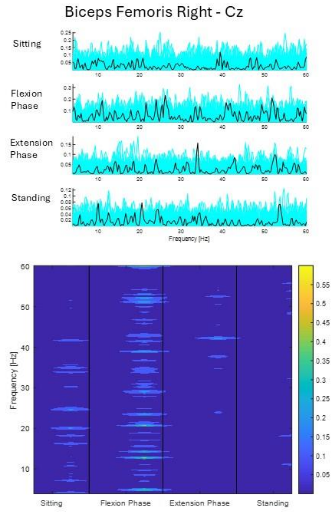
Biceps Femoris Right - Cz
Sitting
Flexion Phase
Extension Phase
Standing
Frequency [Hz]
Frequency [Hz]
Sitting
Flexion Phase
Extension Phase
Standing

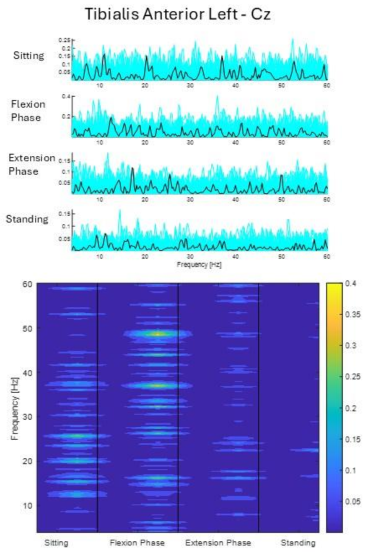
Tibialis Anterior Left - Cz
Sitting
Flexion Phase
Extension Phase
Standing
Frequency [Hz]
Frequency [Hz]
Sitting
Flexion Phase
Extension Phase
Standing

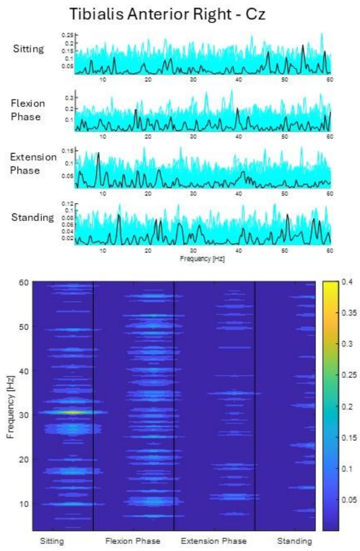
Tibialis Anterior Right - Cz
Sitting
Flexion Phase
Extension Phase
Standing
Frequency [Hz]
Frequency [Hz]
Sitting
Flexion Phase
Extension Phase
Standing

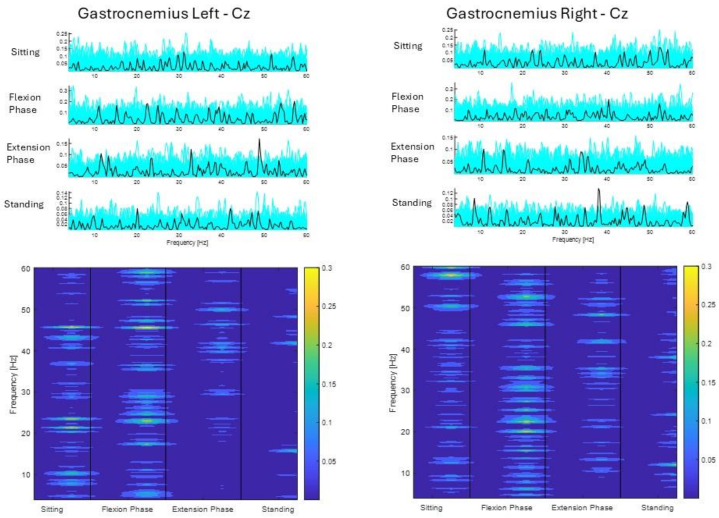
Gastrocnemius Left - Cz
Gastrocnemius Right - Cz
Sitting
Flexion Phase
Extension Phase
Standing
Frequency [Hz]
Sitting
Flexion Phase
Extension Phase
Standing
